# Uncoupled biological and chronological aging of neutrophils in cancer promotes tumor progression

**DOI:** 10.1136/jitc-2021-003495

**Published:** 2021-12-07

**Authors:** Laura A Mittmann, Florian Haring, Johanna B Schaubächer, Roman Hennel, Bojan Smiljanov, Gabriele Zuchtriegel, Martin Canis, Olivier Gires, Fritz Krombach, Lesca Holdt, Sven Brandau, Thomas Vogl, Kirsten Lauber, Bernd Uhl, Christoph A Reichel

**Affiliations:** 1 Department of Otorhinolaryngology, LMU München, Munich, Germany; 2 Walter Brendel Centre of Experimental Medicine, LMU München, Munich, Germany; 3 Department of Radiotherapy and Radiation Oncology, LMU München, Munich, Germany; 4 Institute for Laboratory Medicine, LMU München, Munich, Germany; 5 Department of Otorhinolaryngology, University Hospital Essen, Essen, Germany; 6 Institute for Immunology, University of Munster, Munster, Germany

**Keywords:** head and neck neoplasms, immunity, cellular, immunity, innate, inflammation, neutrophil infiltration

## Abstract

**Background:**

Beyond their fundamental role in homeostasis and host defense, neutrophilic granulocytes (neutrophils) are increasingly recognized to contribute to the pathogenesis of malignant tumors. Recently, aging of mature neutrophils in the systemic circulation has been identified to be critical for these immune cells to properly unfold their homeostatic and anti-infectious functional properties. The role of neutrophil aging in cancer remains largely obscure.

**Methods:**

Employing advanced in vivo microscopy techniques in different animal models of cancer as well as utilizing pulse-labeling and cell transfer approaches, various *ex vivo*/*in vitro* assays, and human data, we sought to define the functional relevance of neutrophil aging in cancer.

**Results:**

Here, we show that signals released during early tumor growth accelerate biological aging of circulating neutrophils, hence uncoupling biological from chronological aging of these immune cells. This facilitates the accumulation of highly reactive neutrophils in malignant lesions and endows them with potent protumorigenic functions, thus promoting tumor progression. Counteracting uncoupled biological aging of circulating neutrophils by blocking the chemokine receptor CXCR2 effectively suppressed tumor growth.

**Conclusions:**

Our data uncover a self-sustaining mechanism of malignant neoplasms in fostering protumorigenic phenotypic and functional changes in circulating neutrophils. Interference with this aberrant process might therefore provide a novel, already pharmacologically targetable strategy for cancer immunotherapy.

## Background

Neutrophils represent the prevailing leukocyte subset in the blood of most mammals.^1^ Besides their prominent role in host defense, these immune cells have recently been implicated in the pathogenesis of malignant tumors. Whereas neutrophils have initially been reported to eliminate cancer cells,^2^ previously published data also point to a protumorigenic potential of these immune cells. In this regard, neutrophils release specific molecular factors that support initiation (*eg,* reactive oxygen species, proteases),^3 4^ proliferation (*eg,* neutrophil elastase (NE)),^5^ angiogenesis (*eg,* matrix metalloproteinase 9 (MMP-9), vascular endothelial growth factor (VEGF)), immune evasion (*eg,* arginase-1),^6^ and metastasis (*eg,* neutrophil extracellular traps)^7^ of tumors. With respect to these divergent findings, the existence of antitumorigenic (‘N1’) and protumorigenic (‘N2’) neutrophil phenotypes has been proposed and is defined by distinct cell surface protein signatures.^8^

Neutrophils are short-lived cells that exhibit an average lifespan in the circulation of 6–12 hours, notwithstanding that longer lifetimes of these immune cells (up to 5.4 days) are subject of controversial discussions.^9^ Under homeostatic conditions, mature neutrophils undergo dramatic age-related changes in their molecular repertoire when chronologically (referring to the progressive passing of the actual amount of time after their release from the bone marrow) aging in the circulation. The resulting phenotypic and functional alterations in these immune cells have been termed ‘biological aging’ of neutrophils. This includes the upregulation of the cell surface chemokine receptor CXCR4 that allows ‘aged’ neutrophils to home back to bone marrow, liver, and spleen *via* the chemokine CXCL12/SDF-1α.^10^ Subsequently, these aged immune cells are cleared by resident macrophages, in turn releasing signals that adjust size and function of the hematopoietic stem cell niche and control granulopoiesis.^11^ In addition to the pivotal role of neutrophil aging in homeostasis, this fundamental biological process has recently been identified to be required for neutrophils to fully develop their anti-infectious functions.^12 13^ To this end, neutrophils employ an autocrine, CXCL2-CXCR2-mediated mechanism that facilitates the release of their granular protein content.^14^ With respect to the potential of malignant tumors to produce such cytokines and to the protumorigenic properties of distinct granular proteins (*eg,* NE, MMP-9) of neutrophils, we hypothesize that aging of these immune cells in the circulation contributes to the progression of cancer.^15^

## Methods

A detailed description of methods is provided in online supplemental information.

10.1136/jitc-2021-003495.supp1 Supplementary data

### Anesthesia

During all surgical and experimental procedures, animals were anesthetized using ketamine (100 mg/kg; Zoetis, Parsippany, New Jersey, USA) and xylazine (10 mg/kg; Bayer, Leverkusen, Germany).

### Animals

Male C57BL/6NCrl, male C3H/HeNCrl, and female Balb/C mice were purchased from Charles River (Sulzfeld, Germany) aging 6–8 weeks (body weight of 15–18 g). Animals were housed under standard conditions (22°C±2°C, 30%–60% humidity, 12 hours light/dark cycle, lights on at 7 am) with access to food and water *ad libitum*.

### Cell lines

A mouse squamous cell carcinoma cell line (SCC VII) and a mammary carcinoma cell line (4T1) were obtained from KL (Department of Radiotherapy and Radiation Oncology, LMU München, Munich, Germany). Cells were cultured in Roswell Park Memorial Institute (Thermo Fisher Scientific, Waltham, Massachusetts, USA) media, supplemented with 10% Fetal Bovine Serum (FBS) (Biochrom, Berlin, Germany) and 1% 4-(2-hydroxyethyl)-1-piperazineethanesulfonic acid (HEPES) (PromoCell, Heidelberg, Germany) at 37°C and 5% CO_2._ Mouse endothelial cells (bEnd.3) were purchased from American Type Culture Collection (Manassas, Virginia, USA) and cultured in DMEM (ATCC) supplemented with 10% FBS.

### Experimental procedures

The trafficking of neutrophils to malignant tumors was analyzed in orthotopic syngeneic mouse models of 4T1 breast cancer (in female BALB/c mice) and of SCC VII head and neck squamous cell carcinoma (in male C3H mice) by multi-channel flow cytometry. Aged and non-aged neutrophils were differentiated by pulse-labeling with BrdU. In heterotopic models of these malignant tumors in the mouse auricle, interactions of neutrophils in the tumor microvasculature were further evaluated by multi-channel *in vivo* fluorescence microscopy. Responses of aged and non-aged neutrophils were differentiated by employing an adoptive cell transfer method.

To characterize the mechanisms underlying the trafficking of aged neutrophils in more detail, confocal microscopy and multiplex ELISA analyses as well as a mouse peritonitis assay and multi-channel *in vivo* fluorescence microscopy on the mouse cremaster muscle were employed. Phenotypic and functional properties of aged and non-aged neutrophils were assessed by multi-channel flow cytometry and different *in vitro* assays (cell activation, cell proliferation) using primary mouse neutrophils.

### Statistics

Data analysis was performed with the statistical software SigmaPlot for Windows (Jandel Scientific, Erkrath, Germany). After confirming normality and equal variance of data (using the Shapiro-Wilk and Brown-Forsythe tests), the one-way analysis of variance (ANOVA) test followed by the Dunnett test (>2 groups) or the t test (two groups) was used for the estimation of stochastic probability in intergroup comparisons. If normality and/or equal variance testing failed, the Kruskal-Wallis one-way ANOVA of ranks test followed by the Dunnett test (>2 groups) or the Mann-Whitney rank-sum test (two groups) was used. For survival estimation, the log-rank test was employed. Mean values and SEM are given. P values<0.05 were considered significant. A detailed report of the statistical analyses employed is provided in online supplemental file 2.

10.1136/jitc-2021-003495.supp2 Supplementary data

## Results

### Gene expression of FPR1 and CXCR4 positively correlates with advanced tumor stages in human cancer

With respect to the capability of malignant tumors to produce cytokines promoting neutrophil aging and to the protumorigenic properties of distinct granular proteins of neutrophils, we hypothesize that aging of these immune cells in the circulation contributes to the progression of cancer. To prove this hypothesis, we first analyzed RNA microarray data from the METABRIC human breast cancer cohort (online supplemental figure S1A).^16^ Here, we found a significant positive correlation between the RNA expression levels of formyl peptide receptor 1 (FPR1), an established marker gene of neutrophils, and of CXCR4, whose gene product increases during neutrophil aging on the surface of these immune cells,^10^ with higher tumor stages. Conversely, RNA expression levels of CXCR2, whose gene product expression decreases during neutrophil aging on the surface of these immune cells,^10^ were slightly attenuated in advanced tumor stages (online supplemental figure S1B). Consequently, overall survival of patients with breast cancer with tumors exhibiting high RNA expression of FPR1 or CXCR4 was more impaired than that of patients with low tumor RNA expression of these molecules (online supplemental figure S1C). These bulk data suggest that neutrophil biological aging might promote cancer progression.

### Tumor-released chemokines uncouple biological from chronological aging of circulating neutrophils in cancer

**Figure 1:**
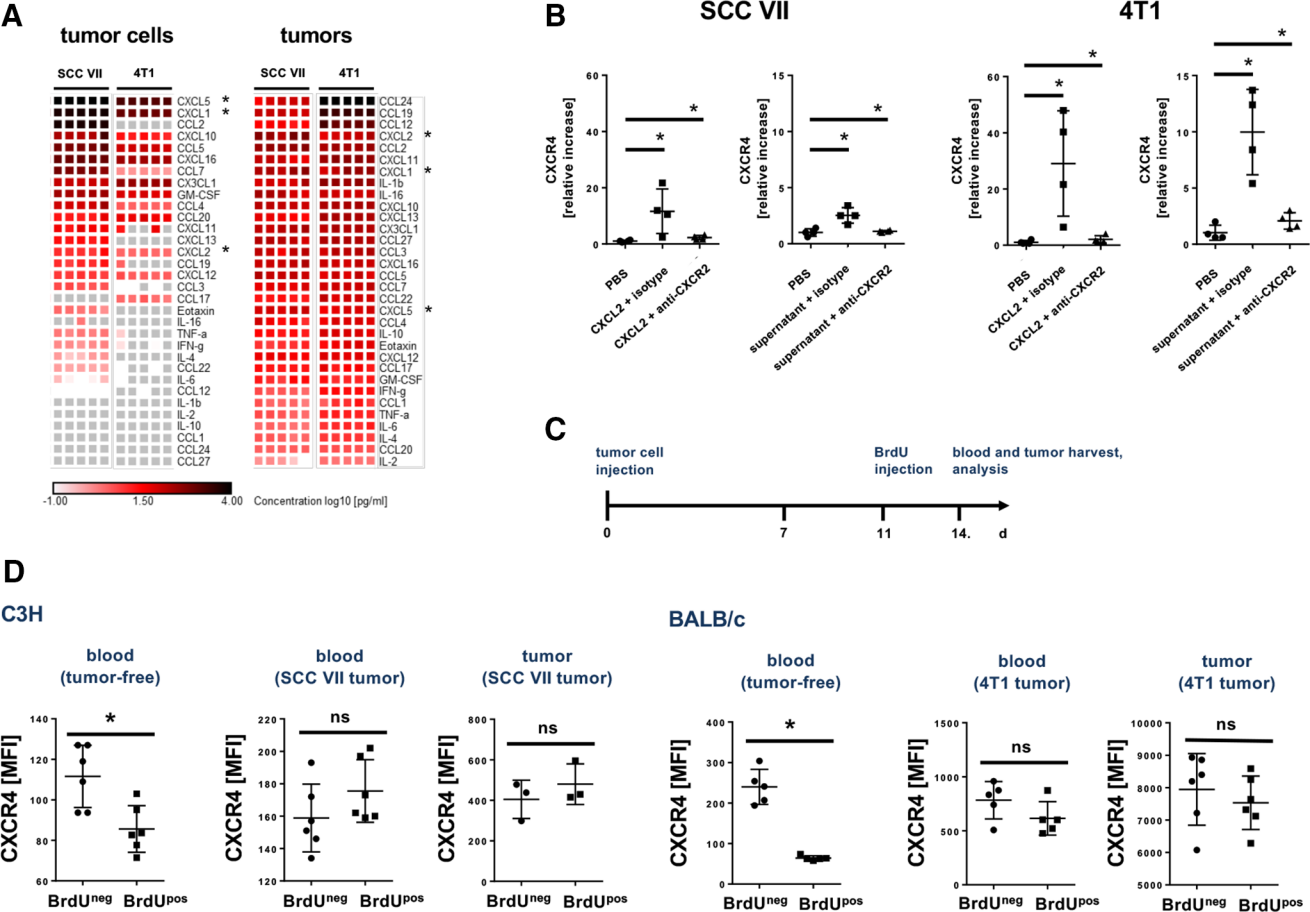
Neutrophil aging in tumor-bearing mice. (A) Expression patterns of different cytokines in SCC VII or 4T1 tumor cell supernatants and orthotopically derived tumors in C3H or BALB/c mice as assessed by multiplex ELISA analyses, data are presented as heatmaps (n=5, each square represents one experiment). Chemokines are listed from top rows (high) to bottom rows (low) according to their expression. Ligands of the chemokine receptor CXCR2 are marked by (*). (B) Relative surface expression of the chemokine receptor CXCR4 on total neutrophils in the peripheral blood of tumor-free C3H or BALB/c mice exposed to the chemokine CXCL2 or supernatant of SCC VII or 4T1 tumor cells on application of blocking anti-CXCR2 or isotype control antibodies as assessed by multi-channel flow cytometry (n=4 mice per group). (C) A schematic illustration of the experimental protocol of BrdU pulse-labeling experiments is shown. (D) Expression of the chemokine receptor CXCR4 on chronologically aged (BrdU^neg^) and non-aged (BrdU^pos^) neutrophils in the peripheral blood of tumor-free or of tumor-bearing C3H or BALB/c mice as well as of those recruited to tumors as assessed by multi-channel flow cytometry (n=3–6 mice per group). Data are shown as mean±SEM; *p<0.05 vs BrdU^neg^/isotype control; ns, not significant; SCC, squamous cell carcinoma.

Since expression of CXCR2 and CXCR4 is not restricted to neutrophils, we sought to directly explore the aging process of circulating neutrophils in animal models of cancer. Toward a more general biological conception, we employed orthotopic, syngeneic mouse models of poorly immunogenic (SCC VII; head and neck squamous cell carcinoma (HNSCC)) and higher immunogenic (4T1 breast cancer) cancer at early, premetastatic stages. Multiplex ELISA analyses revealed distinct expression profiles of cytokines in the cell culture supernatants of these malignant tumor cell lines (figure 1A). Whereas these differences in the cytokine expression patterns were less pronounced in the corresponding lysate of solid neoplasms raised in mice, the amount of the released cytokines was essentially higher. Here, particularly ligands of the chemokine receptor CXCR2 including the chemokine CXCL2, which is known to promote biological aging of neutrophils,^13^ were detected. Accordingly, exposure to CXCL2 or to the supernatant of SCC VII or 4T1 tumor cells induced a significant increase in the surface expression of CXCR4 on neutrophils isolated from the peripheral blood of wild-type (WT) mice (figure 1B). This increase was significantly diminished on antibody blockade of CXCR2.

**Figure 2:**
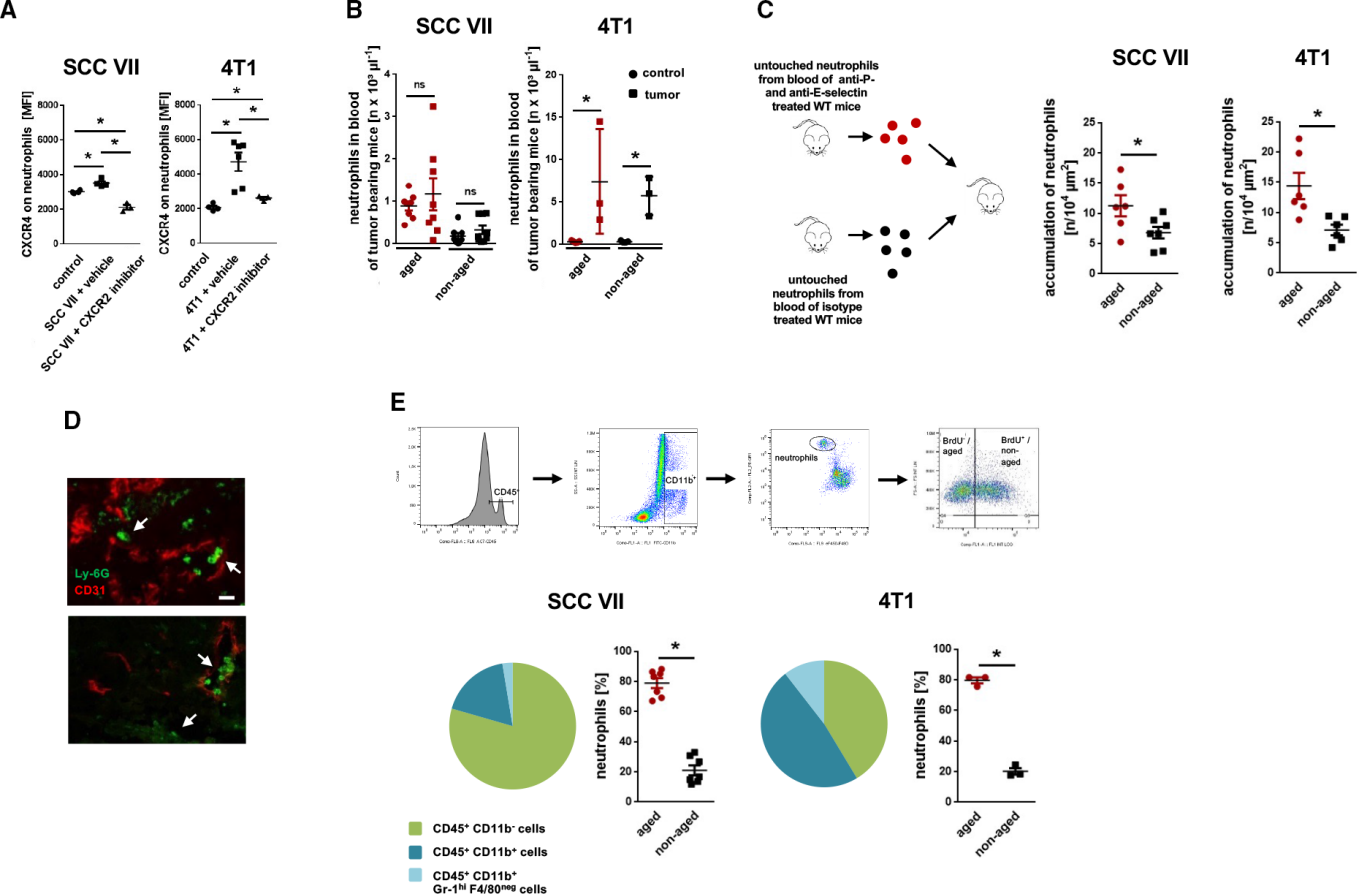
Trafficking of neutrophils in tumor-bearing mice. (A) Surface expression of CXCR4 on total neutrophils in the peripheral blood of tumor-free mice or of tumor-bearing C3H or BALB/c mice treated with a CXCR2 inhibitor or vehicle as assessed by multi-channel flow cytometry (n=3 mice per group). (B) Numbers of circulating aged (BrdU^neg^) and non-aged (BrdU^pos^) neutrophils in tumor-free or tumor-bearing C3H or BALB/c mice, as assessed by multi-channel flow cytometry (n=3–8 mice per group). (C) Accumulation of *ex vivo* fluorescence-labeled aged and non-aged neutrophils in the microvasculature of heterotopically raised tumors in C3H or BALB/c mice, as assessed by *in vivo* microscopy using a cell transfer technique (n=6–7 mice per group). (D) Representative confocal microscopy images of Ly-6G^pos^ neutrophils^32^ and CD31^pos^ microvessels (red) in tissue sections of orthotopically raised tumors in C3H or BALB/c mice are shown (bar: 25 µm; arrows point to neutrophils). (E) Accumulation of leukocytes in tumors as assessed by multi-channel flow cytometry. The gating strategy, the composition of the immune cell infiltrate, and quantitative data for accumulated aged neutrophils (BrdU^neg^) and non-aged neutrophils (BrdU^pos^) in tumors are shown (n=3–7 mice per group). Data are shown as mean±SEM; *p<0.05; ns, not significant; SCC, squamous cell carcinoma; WT, wild type.

To analyze effects of these tumor-released signals on biological aging of circulating neutrophils, we subsequently evaluated the surface expression of the chemokine receptors L-selectin/CD62L, CXCR2, and CXCR4 (which gradually increase (CXCR4) or decrease (L-selectin/CD62L, CXCR2) with biological aging of these leukocytes in the systemic circulation under homeostatic conditions)^12 13 17 18^ on blood neutrophils in SCC VII or 4T1 tumor-bearing mice. Metabolic pulse labeling with BrdU further allowed us to determine the relative chronological age of these immune cells (figure 1C). In healthy tumor-free mice, cell surface expression of CXCR4 was significantly higher in chronologically aged (BrdU^neg^) than in non-aged (BrdU^pos^) blood neutrophils (figure 1D), whereas cell surface expression of L-selectin/CD62L and CXCR2 was significantly decreased (online supplemental figure S2). Most interestingly, expression of CXCR4, CXCR2, and L-selectin/CD62L (figure 1D, online supplemental figure S2) did not significantly differ between chronologically aged (BrdU^neg^) and non-aged (BrdU^pos^) circulating as well as tumor-associated neutrophils in tumor-bearing mice, suggesting that in early stages of cancer chronological and biological aging of neutrophils in the systemic circulation is uncoupled. Moreover, the average expression of CXCR4 was significantly elevated on total circulating neutrophils in tumor-bearing mice compared with total circulating neutrophils in healthy controls (figure 2A). Inversely, the average expression of L-selectin/CD62L and CXCR2 on total circulating neutrophils was significantly diminished in tumor-bearing mice compared with controls (online supplemental figure S3). However, the proportion of chronologically aged and non-aged neutrophils of total neutrophils as detected by BrdU pulse labeling did not vary between tumor-free and tumor-bearing animals (figure 2B), further indicating that in early cancer biological aging of circulating neutrophils is accelerated. Importantly, all neutrophils recruited to the tumors were mature (as indicated by normal cell size and segmented nuclear morphology; online supplemental figure S4), whereas only a small proportion of non-aged (BrdU^pos^) neutrophils in the peripheral blood of tumor-bearing animals exhibited band nuclei (indicative for less neutrophil maturity). This elevation in CXCR4 expression of neutrophils in tumor-bearing animals was almost completely abolished on antibody blockade of the aging-promoting chemokine receptor CXCR2 (figure 2A). Noteworthy, serum levels of CXCR2 ligands only slightly, but not significantly varied between tumor-bearing and tumor-free mice (online supplemental figure S5), pointing to activity of these chemokines in the immediate tumor microvasculature. Collectively, our data suggest that tumor-released CXCR2 ligands uncouple biological from chronological aging of circulating neutrophils in early cancer by promoting excessive biological aging of these immune cells.

### Excessive aging of neutrophils promotes their migration to malignant tumors

To directly elucidate the fate of excessively aging neutrophils in cancer, we isolated aged neutrophils from tumor-free mice treated with blocking anti-P-selectin and anti-E-selectin antibodies which effectively interferes with the elimination of chronologically (and biologically) aged neutrophils in bone marrow, liver, and spleen, thereby enriching BrdU^neg^ CXCR4^hi^ (aged) neutrophils in the systemic circulation (90.2%±2.6% aged neutrophils of total neutrophils). In addition, we isolated non-aged neutrophils from tumor-free mice treated with isotype control antibodies in which most circulating aged neutrophils are physiologically eliminated,^12 13 17 18^ resulting in only 21.6%±1.5% of BrdU^neg^ CXCR4^hi^ aged neutrophils of total neutrophils. After differential *ex vivo* fluorescence labeling and subsequent adoptive transfer of these two different cell fractions into recipient mice heterotopically bearing SCC VII or 4T1 tumors, multi-channel *in vivo* microscopy unveiled that particularly aged neutrophils accumulate in the tumor microvasculature (figure 2C). Immunostaining and confocal microscopy on tissue sections from orthotopic 4T1 and SCC VII tumors further demonstrated a robust infiltration of neutrophils into the malignant neoplasms (figure 2D). The majority of these neutrophils was represented by chronologically aged (BrdU^neg^) neutrophils as evidenced by multi-channel flow cytometry analyses of the tumor lysates (figure 2E). These data indicate that excessive biological aging of neutrophils supports the migration of these immune cells to malignant tumors.

### Activation of the NLRP3 inflammasome by tumor-released DAMPs induces the trafficking of excessively aging neutrophils to malignant tumors

**Figure 3:**
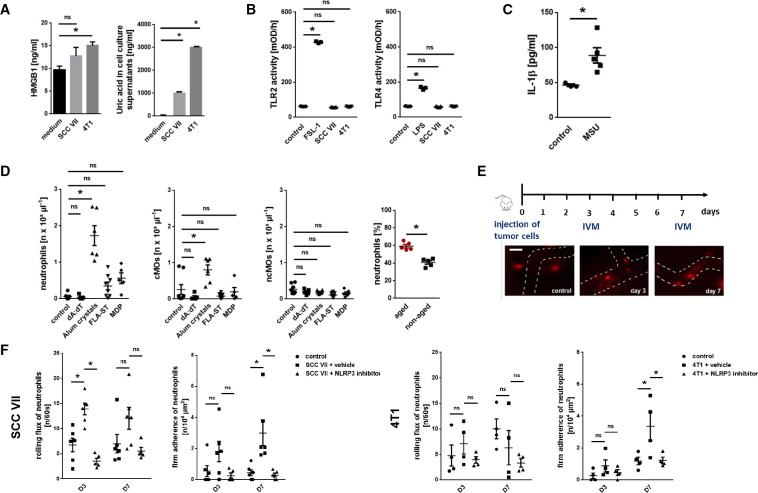
Mechanisms underlying the trafficking of excessively aged neutrophils to malignant tumors. (A) Concentration of HMGB1 or uric acid in tumor cell supernatants, as assessed by ELISA and an automated analyzer (n=4–5 experiments per group). (B) TLR2 and TLR4 receptor activity, as assessed in reporter cells after exposure to SCC VII or 4T1 tumor cell supernatants, medium, or LPS/FSL-1 (n=3–5 experiments per group). (C) IL-1β release from peritoneal macrophages on exposure to MSU, as assessed by multiplex ELISA analysis (n=5 mice per group). (D) Leukocyte recruitment to the peritoneal cavity 6 hours after intraperitoneal injection of different inflammasome-activating substances, as assessed by multi-channel flow cytometry (n=4–8 mice per group). Absolute cell numbers as well as the proportion of aged and non-aged neutrophils of all neutrophils (for stimulation with alum crystals) recruited to the peritoneal cavity are shown. (E) Neutrophil responses in the tumor microvasculature, as assessed by *in vivo* microscopy using heterotopic tumor models of SCC VII or 4T1 in C3H or BALB/c mice. Experimental protocol and representative images (bar: 25 µm) are shown. (F) Neutrophil responses in tumor-bearing mice treated with an NLRP3 inflammasome inhibitor or vehicle are shown (n=4–6 mice per group). Quantitative data are shown as mean±SEM; *p<0.05; ns, not significant. cMO, classical monocytes; IL, interleukin; MSU, monosodium urate; SCC, squamous cell carcinoma; IVM, intravital microscopy; MDP, muramyl dipeptide; TLR, toll-like receptor; LPS, lipopolysaccharide; FSL, fibroblast-stimulating peptide; FLA-ST, Flagellin from Salmonella Typhimurium.

Damage-associated molecular patterns (DAMPs) such as S100A8/9, high mobility group box 1 (HMGB1), or uric acid are released from damaged cells under a variety of pathological conditions including cancer and have been implicated in leukocyte trafficking.^19^ Consequently, these DAMPs might mediate the recruitment of excessively aging neutrophils in cancer to malignant tumors. In further experiments, we document that intraperitoneal stimulation with monosodium urate (MSU) and—to a lesser degree—with S100A8/9 but not with HMGB1 induces the recruitment of mature neutrophils (as indicated by segmented nuclear morphology of these cells; data not shown) and/or classical monocytes (cMOs), but not of non-classical monocytes (ncMOs), to the peritoneal cavity (online supplemental figure S6A). Importantly, in the supernatant of SCC VII and 4T1 tumor cells, we only detected large amounts of uric acid (figure 3A), whereas HMGB1 levels were low and S100A8/9 was not present (not shown). Exposure of the tumor cell supernatants to (toll-like receptor) TLR-2 or TLR-4 (principal receptors of HMGB1) reporter cells, however, did not induce a positive signal in these cells (figure 3B). In contrast, exposure of MSU potently activated peritoneal macrophages as indicated by a significant increase in the production of interleukin (IL)-1β (figure 3C). Accordingly, we detected strong expression of IL-1β and other inflammatory cytokines such as tumor necrosis factor (TNF) in SCC VII and 4T1 tumors (which include macrophages) grown in mice, but barely in the underlying tumor cells (figure 1A).

DAMP-mediated synthesis of IL-1β and, in turn, of other cytokines is mainly regulated through different multimeric intracellular protein complexes termed inflammasomes.^20^ To identify specific inflammasomes mediating responses of aged neutrophils, we performed another set of experiments. Intraperitoneal application of an activator of the nucleotide oligomerization domain-like receptor pyrin domain-containing-3 (NLRP3; alum crystals) inflammasome, but not of the NLRP1 (muramyl dipeptide), the absent in melanoma 2 (AIM2; dA:dT), or the NLRC4 (flagellin) inflammasomes, elicited significant responses of neutrophils and cMOs, but not of ncMOs (figure 3D). The majority of neutrophils recruited on NLRP3 inflammasome activation was represented by aged (BrdU^neg^) neutrophils.

Employing multi-channel *in vivo* microscopy in our heterotopic models of SCC VII HNSCC and 4T1 breast cancer, we further found a significant increase in numbers of intravascularly rolling (SCC VII) and, later on, firmly adherent (SCC VII and 4T1) neutrophils in the tumor microvasculature over the experimental period of 7 days (figure 3E, F). This increase in numbers of rolling and firmly adherent neutrophils was completely abolished on application of a highly specific NLRP3 inflammasome inhibitor. In addition, neutrophilia in 4T1 tumor-bearing mice was abrogated on NLRP3 inflammasome inhibition (online supplemental figure S6B). Hence, the NLRP3 inflammasome controls the trafficking of excessively aging neutrophils to malignant lesions.

**Figure 4:**
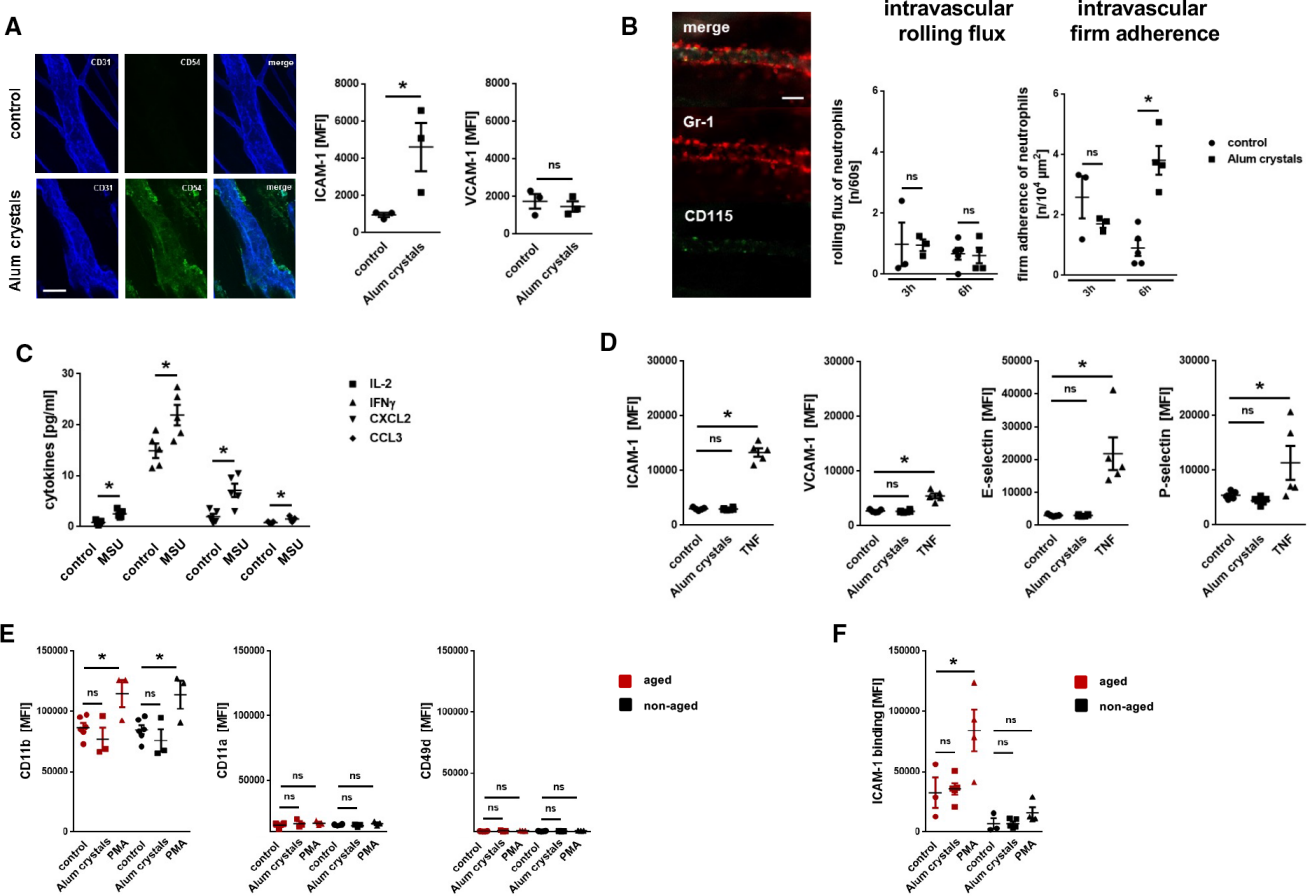
Mechanisms underlying NLRP3 inflammasome-dependent myeloid leukocyte responses. (A) Representative images (bar: 25 µm) and quantitative data for expression of ICAM-1/CD54 and VCAM-1/CD106 on cremasteric endothelial cells on intrascrotal injection of alum crystals or phosphate buffered saline (PBS) (n=3 mice per group). (B) Representative *in vivo* microscopy images of the mouse cremaster muscle (bar: 25 µm) and quantitative data on rolling and adherent neutrophils in postcapillary venules 6 hours after intrascrotal injection of alum crystals or PBS (n=3–5 mice per group). (C) Amount of different cytokines released by peritoneal macrophages on exposure to MSU or PBS as assessed by multiplex ELISA analysis (n=5 mice per group). (D) Expression of ICAM-1/CD54 and VCAM-1/CD106 on bEnd.3 endothelial cells on exposure to alum crystals, TNF, or PBS as assessed by flow cytometry (n=3–5 experiments per group). (E) Expression of different integrins (n=3–5 mice per group) or (F) binding of ICAM-1/CD54-Fc (n=3–5 mice per group) on neutrophils isolated from the peripheral blood of WT mice on exposure to alum crystals, PMA, or PBS as assessed by multi-channel flow cytometry. Data are shown as mean±SEM; *p<0.05. IFN, interferon; IL, interleukin; MSU, monosodium urate; ns, not significant; MFI, mean fluorescence intensity; PBS, phosphate buffered saline; TNF, tumor necrosis factor; VCAM, vascular cell adhesion molecule; ICAM, intercellular cell adhesion molecule; PMA, Phorbol 12-myristate 13-acetate; CCL, chemokine (C-C motif) ligand; CXCL, chemokine (C-X-C) motif ligand.

To further decipher the mechanisms underlying NLRP3 inflammasome-dependent neutrophil responses, we employed more reductionist model systems. Using immunostaining and confocal microscopy on cremasteric tissue whole mounts, we observed a significant increase in the expression of ICAM-1/CD54, but not of VCAM-1/CD106, on endothelial cells of postcapillary venules on intrascrotal stimulation with NLRP3 inflammasome-activating alum crystals compared with unstimulated controls (figure 4). Multi-channel *in vivo* microscopy on the mouse cremaster muscle further revealed a significant increase in numbers of intravascularly adherent, but not of rolling neutrophils, 6 hours after intrascrotal application of alum crystals compared with controls (figure 4B). In this context, activation of the NLRP3 inflammasome in peritoneal macrophages induced the synthesis of IL-1β (and, in turn, of various inflammatory cytokines; figure 4C). However, exposure of alum crystals to microvascular endothelial cells did not induce surface expression of adhesion and signaling molecules—in contrast to the cytokine TNF (figure 4D). Similarly, direct stimulation with PMA, but not with NLRP3 inflammasome-activating alum crystals, elicited surface expression of the integrin CD11b/Mac-1 (but not of CD11a/LFA-1 or VLA-4/CD49d) on neutrophils isolated from the peripheral blood of WT mice (figure 4E). Subsequent binding of ICAM-1/CD54-Fc to aged, but not to non-aged neutrophils, was significantly elevated on exposure to PMA, but not to alum crystals (figure 4F). Thus, activation of the NLRP3 inflammasome in perivascular macrophages induces the synthesis of inflammatory mediators that upregulate adhesion and signaling molecules on the surface of microvascular endothelial cells, in turn promoting the trafficking of aged neutrophils to the perivascular space.

### Excessively aging neutrophils support tumor growth

**Figure 5:**
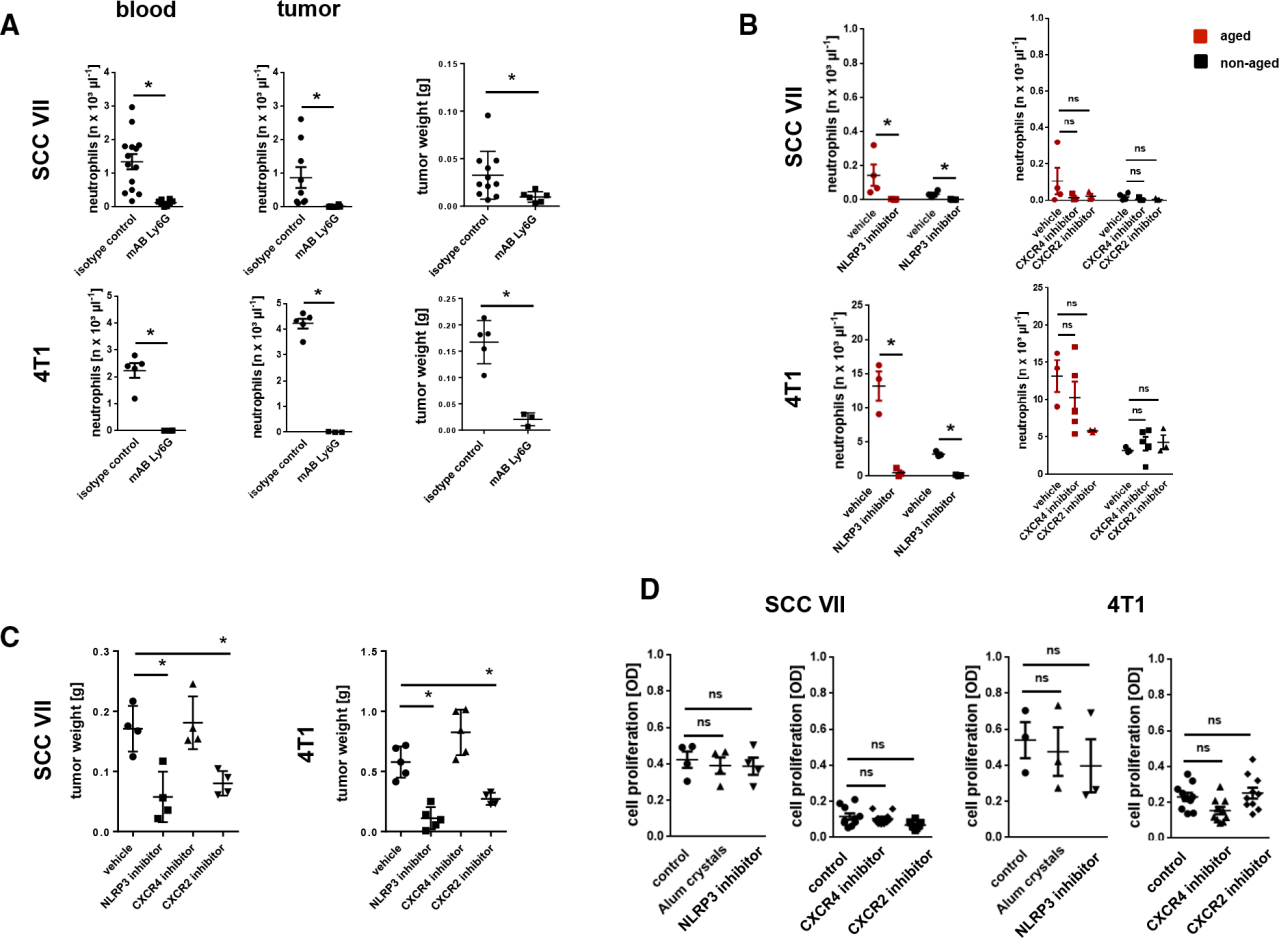
Effect of excessively aging neutrophils on tumor progression. (A) Effects of antibody-mediated neutrophil depletion on neutrophil numbers in peripheral blood or tumors, and on tumor weight, as assessed in orthotopically SCC VII or 4T1 tumor-bearing C3H or BALB/c mice (n=3–14 mice per group). (B) Accumulation of aged (BrdU^neg^) and non-aged (BrdU^pos^) neutrophils in tumors of mice treated with inhibitors of the NLRP3 inflammasome, CXCR2, or CXCR4 or with vehicle, as assessed by multi-channel flow cytometry (n=3–5 mice per group). (C) Tumor weight as assessed in tumor-bearing C3H or BALB/c mice treated with inhibitors of the NLRP3 inflammasome, CXCR2, or CXCR4 or with vehicle (n=4–5 mice per group). (D) Proliferation of SCC VII and 4T1 tumor cells on exposure to inhibitors of the NLRP3 inflammasome, CXCR2, or CXCR4, or to vehicle (n=3–10 experiments per group). Data are shown as mean±SEM; *p<0.05; ns, not significant; SCC, squamous cell carcinoma.

Tumor-bearing animals exhibit excessively aging neutrophils in their peripheral blood (figures 1 and 2, online supplemental figure S2). To characterize the role of these excessively aging neutrophils for tumor progression, we first performed experiments in neutropenic mice which orthotopically developed SCC VII or 4T1 tumors. Antibody-mediated depletion of neutrophils prevented infiltration of the tumors by these immune cells and significantly reduced tumor growth compared with animals treated with isotype control antibodies(figure 5A). In line with our previous results, neutrophil recruitment into the malignant lesions was completely suppressed on blockade of the NLRP3 inflammasome (figure 5B). In contrast, blockade of the aging-promoting chemokine receptor CXCR2 or the aging-inhibiting chemokine receptor CXCR4^13^ did not significantly alter neutrophil migration into the tumors. However, counteracting neutrophil trafficking (by NLRP3 inhibition) or neutrophil aging (by CXCR2 blockade) attenuated tumor progression, whereas supporting neutrophil aging (by blockade of CXCR4) even enhanced tumor growth in SCC VII HNSCC and 4T1 breast cancer (figure 5C). Importantly, direct exposure of NLRP3 activating alum crystals or of the NLRP3 inhibitor as well as of blocking antibodies directed against CXCR2 or of the CXCR4 inhibitor did not significantly change the proliferation of SCC VII or 4T1 tumor cells *in vitro* (figure 5D). Although we cannot exclude that these interventions might additionally alter processes independent from neutrophil biological aging or recruitment, our data strongly suggest that excessively aging neutrophils promote cancer progression.

### Excessively aging neutrophils stimulate cancer cell proliferation via release of NE

Under homeostatic conditions, neutrophils progressively release their protein content from intracellular granules when aging in the circulation. This process initially renders these innate immune cells highly reactive for their fight against invading pathogens, before they ultimately become ‘disarmed’ to prevent unwanted collateral damage.^14^ Extending these observations, the granularity of chronologically aged (BrdU^neg^) neutrophils in the peripheral blood was slightly lower than that in non-aged (BrdU^pos^) neutrophils in both tumor-free and and tumor-bearing animals (online supplemental figure S7A). Most interestingly, these differences were dramatically enhanced in neutrophils isolated from malignant tumors, strongly suggesting that particularly aging tumor-associated neutrophils release their protein content.

**Figure 6:**
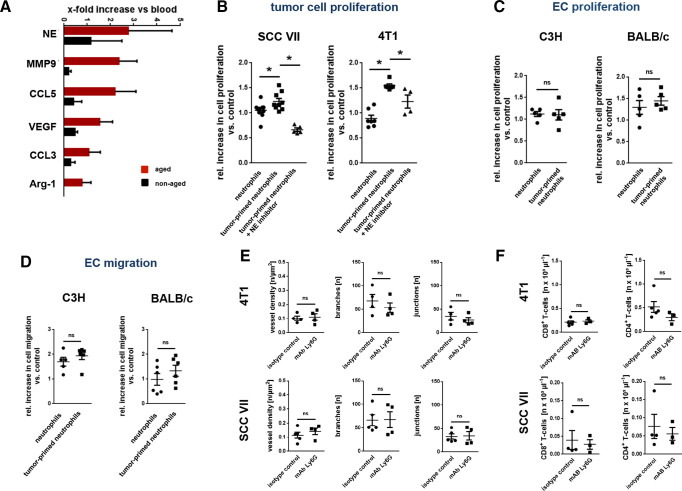
Protumorigenic effects of excessively aging neutrophils. (A) Relative expression levels of NE, MMP9, CCL5, VEGF, CCL3, and arginase-1 on aged and non-aged neutrophils harvested from the peritoneal cavity of WT mice 6 hours after intraperitoneal stimulation with alum crystals, as assessed by multi-channel flow cytometry (n=3–11 mice per group). Effects of blood neutrophils harvested from SCC VII or 4T1 tumor-bearing C3H or BALB/c mice on (B) tumor cell (n=4–10 mice/experiments per group) or (C) brain endothelial cell line 3 (bEnd) endothelial cell (n=5 mice/experiments) proliferation, as assessed *in vitro* by an 3-(4,5-dimethylthiazol-2-yl)-2,5-diphenyl-2H-tetrazolium bromide (MTT) assay. (D) Effects of blood neutrophils harvested from SCC VII or 4T1 tumor-bearing C3H or BALB/c mice on bEnd endothelial cell migration, as assessed in an *in vitro* 2D migration assay (n=5–6 mice/experiments). Architecture of the microvasculature in heterotopic SCC VII or 4T1 tumors, as assessed by *in vivo* microscopy (n=4–5 mice per group). Numbers of CD8^+^ and CD4^+^ T cells in tumors of orthotopically SCC VII or 4T1 tumors, as assessed by multi-channel flow cytometry (n=3–5 mice per group). Data are shown as mean±SEM; *p<0.05; ns, not significant. MMP9, matrix metalloproteinase 9; NE, neutrophil elastase; VEGF, vascular endothelial growth factor; WT, wild-type.

To further define the protumorigenic potential of excessively aging neutrophils, we determined the expression of key molecules supposed to mediate antitumorigenic and protumorigenic effects of these immune cells.^8^ Employing multi-channel flow cytometry on neutrophils isolated from the peripheral blood of mice, we primarily detected higher surface levels of NE, but also of MMP-9, VEGF, CCL3, CCL5, and arginase-1 on aged neutrophils compared with non-aged neutrophils(figure 6A), complementing recent data on enhanced MMP-9 release and NE activity in aged neutrophils compared with non-aged neutrophils.^21^ Furthermore, neutrophils in tumors exhibited slightly higher NE surface levels than neutrophils in the peripheral blood (online supplemental figure S7B). Accordingly, exposure of the supernatant of excessively aging blood neutrophils isolated from tumor-bearing mice to SCC VII HNSCC or 4T1 breast cancer cells (figure 6B), but not to mouse microvascular endothelial cells (figure 6C), induced a significant increase in the proliferation of these tumor cells *in vitro* compared with non-aged and physiologically aged neutrophils isolated from tumor-free mice. This increase was significantly reduced on application of an NE inhibitor (figure 6B).

### Excessively aging neutrophils do not alter tumor angiogenesis or T cell recruitment

Furthermore, migration of microvascular endothelial cells did not significantly differ on exposure to the supernatant of tumor-primed neutrophils or of healthy control neutrophils in an *in vitro* scratch assay (figure 6D). Consequently, antibody-mediated depletion of neutrophils did not significantly alter vessel density as well as the number of branches or junctions in the vessel network of SCC VII or 4T1 tumors as evidenced by multi-channel *in vivo* microscopy in our heterotopic cancer models (figure 6E). Moreover, neutrophil depletion did not significantly alter numbers of CD4^+^ or CD8^+^ T cells in the malignant lesions (figure 6F) as well as the proliferation of these immune cells (determined by Ki-67 immunostaining; online supplemental figure S8), collectively suggesting that excessive aging of neutrophils in cancer particularly promotes NE-dependent tumor cell proliferation.

In tumors derived from the squamous cell carcinoma cell line SCC VII (‘poorly immunogenic’), expectedly less neutrophil (figure 5A) and lymphocyte (figure 6F) responses were observed compared with tumors derived from the breast cancer cell line 4T1 (‘higher immunogenic’). In addition, neutrophil counts were higher in the peripheral blood of (particularly orthotopically) 4T1 tumor-bearing animals than those of SCC VII tumor-bearing animals (online supplemental table S1). These differences in immunogenicity might be explained*—inter alia*—by the different chemokine expression patterns of 4T1 and SCC VII tumors including the chemokine CCL24 (attracting eosinophils, neutrophils, and T cells), the chemokine CCL19 (activating lymphocytes), or the chemokine CCL12 (attracting eosinophils, monocytes, and T lymphocytes; figure 1A). Consequently, all the above reported results were found to be more pronounced in 4T1 tumors than in SCC VII tumors.

## Discussion

Aging of neutrophils in the systemic circulation is critical for the pivotal function of these innate immune cells in homeostasis and host defense against invading pathogens.^11–13^ Recent experimental data in metastatic cancer^21^ and own analyses of the METABRIC human breast cancer cohort, however, suggest that neutrophils exhibiting a phenotype reminiscent of aged neutrophils promote advanced stages of malignant disease. Whether and how neutrophil aging contributes to local tumor growth remains obscure.

To directly evaluate the effect of solid malignancies on aging of circulating neutrophils, we employed orthotopic, syngeneic mouse models of poorly (SCC VII HNSCC) and higher immunogenic (4T1 breast cancer) tumors at early, premetastatic stages.^22^ In the tumor cell culture supernatants and the corresponding solid tumors raised in mice, we identified distinct expression patterns of cytokines in SCC VII and 4T1. Among these molecular factors, particularly ligands of the chemokine receptor CXCR2, including the chemokine CXCL2, were found to be enriched in both SCC VII and 4T1. Previously, CXCL2 has been reported to mediate phenotypic and functional changes related to chronological aging of neutrophils (‘biological aging’) under homeostatic conditions in an autocrine manner,^13^ in addition to its well-known chemotactic properties.^23^ We therefore hypothesized that these tumor-released molecular signals promote such age-related changes in circulating neutrophils.

To prove this hypothesis, we isolated neutrophils from the peripheral blood of tumor-bearing or tumor-free mice and determined cell surface expression levels of CXCR4, which are known to robustly increase with chronological cell aging under both homeostatic and inflammatory conditions (whereas expression levels of CD62L/L-selectin and CXCR2 decrease).^12 18^ As opposed to healthy tumor-free animals, however, expression of CXCR2, CXCR4, and CD62L/L-selectin did not significantly vary between chronologically aged and chronologically non-aged neutrophils in tumor-bearing mice, indicating that in early cancer biological aging of circulating neutrophils is uncoupled from chronological aging. Importantly, the overall expression of CXCR4 on blood neutrophils from tumor-bearing mice was significantly higher compared with non-diseased animals, whereas the proportion of chronologically aged neutrophils of total neutrophils remained stable. These data further point to excessive biological aging of neutrophils in cancer. In line with these results, the elevation in the expression of CXCR4 on circulating neutrophils in tumor-bearing mice was substantially reduced on blockade of the aging-promoting chemokine receptor CXCR2, collectively suggesting that CXCR2 ligands released by malignant tumors uncouple biological from chronological aging of neutrophils in the systemic circulation by promoting excessive biological aging of these immune cells. Notably, serum levels of CXCR2 ligands did not grossly differ between tumor-bearing and tumor-free mice in these early disease states which might be due to cytokine buffering activity of erythrocytes.^24^ Consequently, circulating neutrophils might encounter higher local concentrations of these tumor-released chemokines when passing the tumor microenvironment, where these immune cells are thought to be retained for an extended period of time.^25 26^

Chronological aging of neutrophils induces considerable changes in their molecular repertoire that enable them to rapidly migrate to the site of injury or infection.^12^ The fate of excessively (biologically) aged neutrophils in cancer remains elusive. *In vivo* microscopy in the heterotopic mouse models of HNSCC and breast cancer demonstrated that preferentially adoptively transferred aged neutrophils accumulate in the tumor microvasculature of tumor-bearing recipient mice. In line with these observations, metabolic pulse-labeling experiments in the orthotopic tumor models further revealed that particularly neutrophils with a high relative chronological age infiltrate and reside in malignant tumors. Hence, aging of circulating neutrophils in cancer effectively supports the recruitment of these immune cells into malignant tumors. These findings might be explained by previous reports documenting that aged neutrophils are highly reactive due to increased surface levels of integrins in high affinity confirmation on these immune cells facilitating endothelial interactions in the inflamed microvasculature.^12 18^

Chemokine production varies widely between different cancer entities including SCC VII and 4T1 tumors. DAMPs such S100A8/9, HMGB1, or uric acid, however, are constantly released on cell injury under pathological conditions, including oncological disorders.^19^ In our experiments, we found that SCC VII as well as 4T1 cancer cells abundantly produce uric acid and—to a much lesser degree—HMGB1, but not S100A8/A9. In principle, these two detectable DAMPs are able to potently recruit aged neutrophils. However, the supernatants of these tumor cells did not activate TLR-2 or TLR-4 reporter cells, suggesting that HGMB1 (which mediates cell activation primarily via these receptors) is not dominant in regulating neutrophil trafficking to these malignant lesions. Since MSU is known to serve as a potent activator of the NLRP3 inflammasome, facilitating the synthesis of IL-1β and other inflammatory mediators,^20^ we assumed that inflammasome activation in the tumor environment controls the trafficking of excessively aging neutrophils to malignant tumors. In line with this assumption, activation of the NLRP3 inflammasome potently elicited responses of neutrophils in reductionist *in vivo* assays. Mechanistically, NLRP3 stimulation promoted neutrophil–endothelial cell interactions *via* the production of various cytokines and chemokines in macrophages capable of activating microvascular endothelial cells, but did neither directly activate endothelial cells nor neutrophils. Consequently, application of a highly specific NLRP3 inflammasome inhibitor almost completely abrogated neutrophilia and accumulation of excessively aging neutrophils in malignant tumors, but did not alter biological aging of these immune cells as indicated by unchanged surface expression levels of CXCR4. Taken together, our data suggest that activation of the NLRP3 inflammasome in (peritumoral) macrophages promotes the trafficking of excessively aging neutrophils to malignant lesions. Importantly, different malignancies exhibit specific spatiotemporal activation patterns of inflammasomes in tumor cells and their environment which might cause the previously published multifaceted (and also controversial) functions of this intracellular protein complex in different tumor entities.^27–29^ Specifically, the enormous phenotypic and functional heterogeneity of macrophages and endothelial cells in different tissues, pathologies, and disease states might contribute to these divergent results.

Next, we sought to characterize the effect of excessively aging neutrophils on the progression of malignant tumors. We found that antibody-mediated depletion of these immune cells significantly reduces the growth of tumors in experimental HNSCC and breast cancer. Similar results were obtained on inhibition of NLRP3 inflammasome-dependent neutrophil infiltration of these malignant lesions, indicating that excessively aging neutrophils support tumor growth. Although initial studies were not able to delineate a functional role of neutrophils in cancer models,^30^ our findings are in line with the majority of previously published experimental work reporting tumor-promoting properties of neutrophils.^31 32^ Accordingly, chemotherapy-induced neutropenia in patients with cancer was found to be associated with a favorable disease outcome,^33^ whereas application of granulocyte-colony stimulating factor to neutropenic individuals^34^ as well as high neutrophil-lymphocyte ratios in the peripheral blood or high neutrophil numbers in tumors^35^ are related to poor survival rates in oncological disorders. Noteworthy, aged neutrophils have recently been shown to promote thrombosis,^13^ suggesting that these specifically primed immune cells might also contribute to dreaded comorbidities of patients with cancer.^36 37^

To characterize age-related effects of neutrophils on tumor growth in more detail, we directly manipulated the aging process of circulating neutrophils in tumor-bearing mice. Blockade of the CXCL2 receptor CXCR2, which interferes with neutrophil biological aging,^13^ reduced tumor growth and—in line with recent observations^38^ and most probably due to the downregulation of CXCR2 on excessively aging neutrophils—did not significantly affect neutrophil trafficking into malignant lesions or directly modulate tumor cell proliferation. In contrast, blockade of the CXCL12-CXCR4 axis by a CXCR4 inhibitor (which supports the mobilization of non-aged neutrophils from the bone marrow into the systemic circulation, interferes with the recruitment of aged neutrophils back to bone marrow, liver, and spleen as well as directly promotes neutrophil aging^13 39^) even slightly enhanced tumor growth, again without altering neutrophil infiltration of the neoplasms or directly affecting tumor cell proliferation. Thus, our experimental findings indicate that excessive aging of neutrophils in the periphery renders these immune cells protumorigenic. Interestingly, however, inhibition of CXCR4 signaling has recently been reported to interfere with responses of protumorigenic neutrophils in a zebrafish xenotransplantation model of breast cancer^40^ as well as of immunosuppressive Ly6C^low^ monocytes in experimental colorectal cancer,^41^ pointing to distinct roles of this chemokine receptor, which can also be expressed by tumor cells, in different types of cancer.

According to the previously reported antitumorigenic and protumorigenic properties of neutrophils,^8^ the existence of distinct phenotypes of neutrophils has been proposed. Following the classification for macrophages, an antitumorigenic ‘N1’ and a protumorigenic ‘N2’ phenotype have been defined by specific surface protein expression signatures.^42^ We here identified that aging of neutrophils is associated with a more protumorigenic state, as indicated by increased surface expression of NE, MMP-9, VEGF, chemokines CCL3 or CCL5, and arginase-1 on these immune cells. These findings are in line with recent reports on the progressive release of neutrophil granular proteins during physiological aging of these immune cells in the circulation.^14^ Most interestingly, we found that this loss of granularity in aging neutrophils is also present in the circulation of tumor-bearing animals and, moreover, is particularly pronounced in tumor-associated neutrophils. In accordance with these observations, we were able to demonstrate that excessively aging neutrophils potently stimulate the proliferation of tumor cells *in vitro*. These effects were dependent on NE, which has previously been identified to degrade insulin receptor substrate 1 in the endosomal compartment of tumor cells on intracellular uptake of this protease. As a consequence of these events, interactions of phosphoinositide 3-kinase (PI3K) and platelet-derived growth factor receptor increase, thereby skewing the PI3K axis toward tumor cell proliferation. Furthermore, NE is supposed to upregulate mitogen-activated protein kinases, further enhancing the proliferation of tumor cells.^5 43–46^ In contrast, neutrophils from tumor-bearing mice did not significantly alter the proliferation or migration of microvascular endothelial cells *in vitro* and neutrophil-depletion did not affect the formation of the tumor vessel network *in vivo*. These data indicate that excessively aging neutrophils in cancer might be functionally different from the subset of CD49d^high^ VEGFR1^high^ CXCR4^high^ neutrophils identified to support vascularization of non-vascularized hypoxic tissue^47 48^ or MMP9^high^ VEGF^high^ CXCR4^high^ neutrophils that promote angiogenesis in experimental melanoma or fibrosarcoma.^49^ Likewise, lymphocyte infiltration of malignant tumors as well as the proliferation of these immune cells was not affected in the absence of excessively aging neutrophils, collectively suggesting that these protumorigenic neutrophils do not exhibit significant immunosuppressive properties as myeloid-derived suppressor cells, but promote tumor progression through NE-dependent stimulation of tumor cell proliferation.

## Conclusion

Our experimental findings uncover a previously unknown self-sustaining mechanism of malignant tumors in uncoupling biological from chronological aging of circulating neutrophils. In contrast to the beneficial role of neutrophil aging in homeostasis and host defense against invading pathogens, excessive biological aging of these immune cells in cancer endows them with potent protumorigenic properties—a mechanism that is more pronounced in higher than in poorly immunogenic tumors. Interference with this aberrant process effectively compromises the progression of malignancies and might therefore provide a novel strategy for the treatment of oncological disorders. This translational approach seems to be particularly promising since first clinical trials employing inhibitors of the aging-promoting chemokine receptor CXCR2 on neutrophils reported positive results in patients with breast cancer.^50^

## Data Availability

All data relevant to the study are included in the article or uploaded as supplementary information.
