# Supplementary material for: Uncoupled biological and chronological aging of neutrophils in cancer promotes tumor progression
Source: J Immunother Cancer. 2021 Dec 7;9(12):e003495. doi: 10.1136/jitc-2021-003495 (PMC8655594; doi:10.1136/jitc-2021-003495)
Supplement: Supplementary data [file jitc-2021-003495supp001.pdf]

SUPPLEMENTARY INFORMATION

Supplementary Materials and Methods

Analyses of RNA microarray data from the METABRIC breast cancer cohort

For analyses of RNA microarray data of the METABRIC breast cancer cohort [1], CBioportal was employed [2]. The subcohort of patients with documented tumor stage and available RNA microarray data was used (n=1403), and follow-up was cut to 120 months. RNA co-expression analyses were done utilizing Spearman and Pearson algorithms. Survival analyses were performed in subgroups of different tumor stages with the Cox proportional hazard model, and target gene expression z-values of ≥ 2.0 were used as strata.

Animal models

<u>Orthotopic tumor models</u>

Tumor cells (at a concentration of 2 x 10^5 cells/20 µl) were either injected into the floor of the mouth (SCC VII) or into the left mammary fat pad (4T1) of mice. Two weeks after application of tumor cells, tumor tissue and blood were harvested. The tumors were weighed and then homogenized in 15 ml saline before centrifugation, removal of the cell-free supernatant and resuspension in a final volume of 500 µl PBS. Of each sample, 100 µl were then immunostained with anti-CD45 APC-Cy7 (BD Bioscience, San Jose, California, USA), anti-CD11b PerCp-Cy5 (eBioscience, San Diego, California, USA), anti-Gr-1 PE (eBioscience), anti-F4/80 efluor450 (eBioscience), and anti-CXCR4 APC (Biolegend, San Diego, California, USA), and in selected experiments with anti-CD8a PE-Cy7 (eBioscience) and anti-CD4 AF700 mAb (eBioscience) for 30 min on ice. Erythrocytes were lysed using a lysing solution (1:10, BD FACS Lysing solution, BD Bioscience). After two washing steps with PBS, samples were resuspended in 200 µl PBS and analyzed using multichannel flow cytometry (total cell preparation time of about 1h after tumor explantation; cell preparation time for experiments with BrdU of about 2.5h, see below).

Mittmann LA, *et al. J Immunother Cancer* 2021; 9:e003495. doi: 10.1136/jitc-2021-003495

To differentiate between fresh neutrophils and aged neutrophils, a pulse labeling technique with 5-BrdU (FITC BrdU Flow kit, BD Bioscience) was used as described previously [3]. Briefly, in order to label neutrophil precursors in the bone marrow, mice received a single intravenous injection of BrdU (2.5 mg/kg), followed by incubation for 72 h. After harvesting the blood and immunophenotyping the blood cells (see above), FITC BrdU staining (which includes DNase treatment) was performed according to the manufacturer's protocol. As BrdU is incorporated into newly synthesized DNA, chronologically non-aged neutrophils appear BrdU-positive and chronologically aged neutrophils BrdU-negative when analyzed by flow cytometry. Accordingly, chronologically aged ($BrdU^{neg}$) neutrophils exhibit at least a 72 h longer lifetime than chronologically non-aged ($BrdU^{pos}$) neutrophils. In selected experiments, additional DAPI staining was performed for the visualization of the cell nucleus prior to the BrdU detection protocol.

This protocol was also used to assess the effects of treatment with the NLRP3 inflammasome inhibitor MCC950 (10 mg/kg, InvivoGen, San Diego, California, USA), the CXCR2 inhibitor (SB 225002; 5 mg/kg, Tocris, Bristol, England), and the CXCR4 inhibitor (AMD3100; 5 µg/kg, Tocris) on tumor development and neutrophil infiltration of tumors.

Neutrophils were depleted in tumor bearing mice by injecting anti-Ly6G mAb (100 µg, clone 1A8, BioXell, Lebanon, New Hampshire, USA) every 48 h, starting with the day of tumor cell injection. In separate experiments, antibody-mediated neutrophil depletion was performed one week after injection of tumor cells. On day 7 after the onset of neutrophil depletion, blood and tumor tissue were harvested and cells were analyzed by a ProCyte Hematology analyzer (IDEXX, Westbrook, Maine, USA) and flow cytometry.

Heterotopic tumor models

In vivo microscopy analyses of the peritumoral microvasculature were performed in a heterotopic tumor model. For this purpose, tumor cells were injected into the outer ear of C3H or Balb/c mice at a concentration of 2 x 10^5 cells/20 µl. On day 3 or day 7 after injection,

mice ears were placed on a custom-made microscopy stage. Neutrophils were visualized by anti-Ly6G PE mAb (BD Bioscience) applied *via* tail vein injections. This protocol was also used to assess the effects of MCC950 (10 mg/kg) on neutrophil responses in the peritumoral microvasculature.

In adoptive cell transfer experiments, tumor-free wildtype (WT) donor mice were treated with anti-E-selectin (50 µg, BD Biosciences) and anti-P-selectin mAb (50 µg, Biolegend) 48 and 24 h prior to *in vivo* microscopy in order to keep neutrophils within the circulation, thus, leading to enrichment of excessively ageing neutrophils within the circulation. Non-aged neutrophils were obtained from isotype treated tumor-free WT donor mice. Subsequently, whole blood samples were harvested and cleared from red blood cells *via* red blood cell aggregation and sedimentation using HetaSep™ (STEMCELL Technologies, Vancouver, Canada). After labeling neutrophils with differentially fluorescence-labeled anti-Ly6G mAb, anesthetized tumor bearing recipients (day 7 after tumor cell injection) received an intravenous injection of 2 x 10^5 cells (in 20 µl PBS) 30 min before intravital ear imaging.

In order to analyze angiogenesis within the tumor and its microenvironment in neutrophil-depleted tumor bearing mice (day 7 after tumor cell injection) and in isotype control antibody-treated tumor bearing mice, *in vivo* microscopy was performed using a 10x objective after intravenous injection of 50 µl of FITC Dextran (molecular weight 2.000 kDa; Sigma Aldrich, St. Louis, Missouri, USA) to visualize the microvasculature.

M. cremaster assay

The surgical preparation of the mouse cremaster muscle was performed as previously described by Baez with minor modifications [4]. Briefly, the left femoral artery of anesthetized mice was cannulated in a retrograde manner in order to allow administration of antibodies. Next, the right cremaster muscle was exposed through a ventral incision of the scrotum. The muscle was then opened ventrally and spread over a pedestal of a custom-made microscopy stage. After the epididymis and testicle were detached from the cremaster muscle, they were

placed back into the abdominal cavity. Throughout the surgical preparation and *in vivo* microscopy, the muscle was superfused with warm buffered saline.

Leukocyte responses were visualized by *in vivo* microscopy (see below) 3 or 6 h after intra-scrotal injection of Alum crystals (10 µg, InvivoGen). Neutrophils as well as classical and non-classical monocytes were identified after intra-venous injection (see below) of anti-Gr-1 PE (eBioscience) and anti-CD115 AF594 (Biolegend) antibodies.

Peritonitis assay

Leukocyte recruitment to the peritoneal cavity was studied 6 h after induction of peritoneal inflammation. Mice were sacrificed and their peritoneal cavity was washed with 10 ml of ice-cold saline. The total number of leukocytes in the peritoneal lavage fluid was measured with the ProCyte Hematology analyzer (IDEXX, Westbrook, Maine, USA). Samples were then immunostained using antibodies directed against CD45 (APC-Cy7, BD Bioscience), CD11b (FITC, BD Bioscience or PerCp-Cy5, eBioscience), Gr-1 (PE, eBioscience), F4/80 (efluor450, eBioscience), or CXCR4 (APC, Biolegend) for 30 min on ice. After lysing erythrocytes (1:10, BD FACS Lysing solution, BD Bioscience) and two washing steps with PBS, samples were resuspended in 200 µl PBS. In order to investigate the effect of different DAMPs on leukocyte recruitment, HMGB1 (1 µg, Biolegend), S100A8/A9 (1 µg, Biolegend), and MSU crystals (10 µg, InvivoGen) were injected into peritoneal cavity of WT mice.

The same protocol was used to assess the effect of inflammasome activating substances, such as poly da:dt (10 µg, InvivoGen), FLA-ST (10 µg, InvivoGen), MDP (10 µg, InvivoGen,), or Alum crystals (10 µg, InvivoGen). Again, fresh neutrophils and aged neutrophils were identified by BrdU pulse labeling (see above).

Flow cytometry

Employing multi-channel flow cytometry (Gallios, Beckman Coulter Inc, Brea, California USA), myeloid leukocytes were identified by expression of CD45 and CD11b. By the use of Gr-1, F4/80, and CD115, these cells were further divided into neutrophils (Gr-1^{high}, F4/80^{neg}, CD115^{neg}), classical monocytes (Gr-1^{high}, F4/80^{pos}, CD115^{pos}), and non-classical monocytes (Gr-1^{low}, F4/80^{pos}, CD115^{pos}). Lymphoid leukocytes such as T-cells were identified as CD11b^{-} and CD4^{+} or CD8a^{+} cells. Neutrophils were further differentiated into non-aged neutrophils (BrdU^{pos}) and aged neutrophils (BrdU^{neg}), and by the expression of CXCR4. In selected experiments, expression of L-selectin/CD62L (PE; BD Biosciences) in neutrophils or expression of Ki-67 (AF488; Invitrogen) in lymphocytes was measured. All results were quantified with the FlowJo Software (Treestar, Ashland, Oregon, USA).

In vivo microcopy

In vivo microscopy was performed using an AxioTech-Vario 100 Microscope (Zeiss MicroImaging GmbH, Goettingen, Germany), equipped with a Colibiri LED light source (Zeiss MicroImaging GmbH) for fluorescence epi-illumination microscopy. All microscopy videos were obtained with an AxioCam Hsm digital camera using a 40x water immersion lens (0.5 NA, Zeiss MicroImaging Gmbh), and processed with the AxioVision 4.6 software (Zeiss MicroImaging GmbH).

Videos were later analyzed by using the imaging software Fiji [5]. In the heterotopic ear imaging model neutrophils were identified by being Ly6G^{pos}. In the mouse cremaster muscle these immune cells were identified as Gr-1^{pos} CD115^{neg} cells, classical monocytes as Gr-1^{pos} CD115^{pos}, and non-classical monocytes as Gr-1^{neg} CD115^{pos}. Rolling leukocytes were defined as those moving slower than the associated blood flow and quantified for 60 s per venule. Firmly adherent cells were determined as those resting in the associated blood flow for >30 s and related to the luminal surface per 100 µm vessel length. Architecture of the microvasculature was analyzed with the Skeleton plugin. Briefly, by generating skeletonized maps of the vessel network, this plugin is able to determine the number of branches per high

power field, the number of junctions as well as the average branch length. The vessel density was characterized as the number of branches multiplied by the average branch length / area of the high-power field.

Phenotyping of neutrophils

To analyze the phenotype of neutrophils with regard to tumorigenicity, the peritonitis assay after intraperitoneal injection of Alum crystals was performed as described above. First, harvested peritoneal lavage samples were immunostained using antibodies directed against CD45 (APC-Cy7,BD Bioscience), CD11b (PerCp-Cy5, eBioscience), Gr-1 (PE or AF488, eBioscience, or Biolegend), F4/80 (efluor450, eBioscience), CD115 (AF594, Biolegend) and CXCR4 (APC, Biolegend) including further immunostaining either with anti-MMP9 (FITC, StressMarq Biosciences, Victoria, Canada), anti-CCL5 (PE, Biolegend), anti-CCL3 (PE, eBioscience), anti-VEGF (FITC, Novus Biologicals, Centennial, Colorado, USA), anti-Arginase-1 (PE, R&D Systems, Minneapolis, Minnesota, USA), or anti-neutrophil elastase (R&D Systems). Neutrophils were identified as $CD45^+$ $CD11b^+$ $Gr\text{-}1^+$ $CD115^-$ cells which almost exclusively represent $Ly\text{-}6G^+$ cells [6]. For neutrophil elastase immunostaining, a secondary antibody (goat anti-rat AF488, Thermo Fisher Scientific, Waltham, Massachusetts, USA) was used that was incubated for 20 min at RT before lysing the remaining erythrocytes with Lysing solution (1:10, BD FACS Lysing solution), washing, and resuspending the samples. Expression levels on neutrophils were measured *via* multichannel flow cytometry. The same protocol was used to assess the expression levels in anticoagulated blood samples.

Immunohistochemistry and confocal microscopy

Tumors were surgically removed from tumor bearing mice and embedded in Tissue-Tek (Sakura, Alphen am Rhein, Netherlands). After storing the samples at -80 °C, sections were cut at 20 µm using a cryostat (Thermo Fisher Scientific) and mounted onto glass slides

(Thermo Fisher Scientific). Subsequently, sections were fixed with 4 % formaldehyde (Microcos, Garching, Germany) for 10 min at RT, followed by washing the slides in PBS for 10 min. Blocking and permeabilization was achieved by incubating the slides in 2 % BSA (Sigma Aldrich, St. Louis, Missouri, USA) in PBS with 0.001 % Triton X-100 (Sigma Aldrich) for 1.5 h at RT. Finally, sections were labeled with anti-CD31/PECAM AF647 (1:100, Biolegend; identification of vascular endothelial cells) and anti-Ly6G PE (1:50; identification of neutrophils) in blocking solution at 4 °C over night. After washing the slides in PBS for 5 min twice, samples were mounted using PermaFluor (Beckman Coulter, Brea, California, USA) and stored at 4 °C.

In order to investigate expression of ICAM-1/CD54 and VCAM-1/CD106 within cremasteric tissue, excised mouse cremaster muscles (6 h after intrascrotal injection of Alum crystals or saline) were fixed in 4 % paraformaldehyde. Next, tissues were blocked and permeabilized using 2 % BSA in PBS with 0.001 % Triton X-100 for 1.5 h at RT. After immunostaining the whole mounts with antibodies directed against PECAM-1/CD31 AF647 and ICAM-1/CD54 (rat, Biolegend) or VCAM-1/CD106 (rat, Biolegend) in blocking solution at 4 °C over night, two washing steps were performed. Subsequently, secondary staining with a goat anti-rat AF488 antibody (Invitrogen, Carlsbad, California, USA) for another 2 h at RT followed, before the immunostained tissues were washed twice and finally mounted in PermaFluor (Thermo Fisher Scientific) on glass slides.

To assess the nuclear morphology of mouse neutrophils, heparinized blood or tumor lysates were incubated with an Alexa Fluor 594-linked rat anti-mouse Ly-6G mAb (1A8; BioLegend) for 30 minutes on ice. Erythrocytes were then lysed with lysing solution (1:10; BD FACS lysing solution; BD Bioscience) and cells were plated on coverslips. Subsequently, samples were incubated with hoechst nucleic acid stain for 15 minutes.

Confocal z-stacks (z-spacing 0.5 – 1 µm) were acquired using a Leica SP8 confocal laser-scanning microscope (Leica Microsystems, Wetzlar, Germany) with an oil-immersion lens (Leica; 40x; NA 1.40). The fluorescence signal was quantified using the software Fiji. Background signal was subtracted.

HMGB1 and s100A8/A9 ELISA

HMGB1 was measured in tumor cell supernatants using the HMGB1-ELISA kit (IBL International, Hamburg, Germany) according to the manufacturers' protocols.

S100A8/A9 concentrations were determined by a sandwich ELISA system established in Thomas Vogl's laboratory as described previously [7]. Briefly, wells were coated with the capturing biotin-coupled polyclonal antibody anti-S100A8 (4 µg/ml, 50µl/well) and polyclonal anti-S100A9 (0.5 µg/ml, 50 µl/well). As a substrate, Streptavidin coupled HRP enzyme and TMB were used for quantification. Absorbance was measured at 405 nm using an MRX microplate reader (Dynex, Berlin). Purified recombinant heterodimer S100A8/S100A9 served as standard (filled circles; solid line: calculated sigmoidal standard curve).

Uric acid measurements

Uric acid levels in cell culture supernatants were determined by a COBAS 8000 modular analyzer (Roche, Mannheim, Germany).

TLR-2 and TLR-4 reporter assays

The activation of TLR-2 and TLR-4 by tumor cell supernatants was determined by using the HEK-Blue^{TM} TLR reporter cell lines (InvivoGen, Toulouse, France), as described previously (Krombach, et al, 2018). Briefly, 50,000 reporter cells per well were seeded in HEK-Blue^{TM} detection medium into 96-well plates, pre-filled with 20 µl tumor cell supernatants. LPS (10 ng/ml) or fibroblast-stimulating lipoprotein-1 (FSL-1, 10 ng/ml, both from InvivoGen) served as positive controls, respectively. The activity of secreted alkaline phosphatase (SEAP) was measured every 2 min in a Synergy MX plate reader over 10 h at 37°C (absorption at 630 nm, BioTek, Berlin, Germany) and was calculated from the slope of the corresponding regression line.

Mittmann LA, *et al. J Immunother Cancer* 2021; 9:e003495. doi: 10.1136/jitc-2021-003495

Multiplex cytokine ELISAs

Concentration of cytokines in tumors, tumor cell supernatants, and mouse sera were analyzed on a Bio-Plex 200 system, using the Bio-Plex Pro™ Mouse chemokine panel 33-Plex according to the manufacturer's protocol (Bio-Rad laboratories, Munich, Germany). Tumors were lysed with the Bio-Plex® Cell Lysis Kit according to the manufacturer's protocol, and 70 µg tumor protein lysate were subjected to the multiplex analysis. Tumor cell supernatants were used undiluted. Group comparisons were performed by unpaired Student's ttests with subsequent Benjamini-Hochberg correction. FDR q-value < 0.1 was used as cutoff for statistical significance.

Activation of neutrophils

As a measure of neutrophil activation, surface expression of the integrins LFA-1/CD11a, Mac-1/CD11b, and VLA-4/CD49d was determined in anticoagulated blood samples incubated for 30 min either with Alum crystals (10 µg, InvivoGen), TNF (100 µg, R&D Systems), CXCL2 (100 ng, Biolegend), supernatant from SCC VII or 4T1 tumor cells, and PBS as negative control. Subsequently, samples were washed with PBS and cells were labeled with antibodies directed against CD45 (APC-Cy7, BD Bioscience,), CD11b (PerCP-Cy5, eBioscience), Gr-1 (PE, eBioscience), F4/80 (efluor450, eBioscience), CXCR4 (APC, Biolegend), and CD11a (FITC, eBioscience), or CD49d (FITC, eBioscience). Lyses of erythrocytes with lysing solution followed. After washing the samples twice in PBS, samples were resuspended in 200 µl PBS and analyzed by multichannel flow cytometry as described earlier.

As a measure of conformational changes of integrins, binding of ICAM-1/Fc to neutrophils was analyzed. Briefly, blood was taken from WT mice, anticoagulated, and suspended in Hanks balanced salt solution containing 1 mM CaCl2 and MgCl2 (Life Technologies,

Carlsbad, California, USA). Subsequently, cells were incubated with PMA (50 ng/ml, Sigma Aldrich,St. Louis, Missouri), Alum crystals (10 µg, InvivoGen), or PBS as negative control for 30 min at 37 °C , followed by adding ICAM-1/Fc (10 µg/ml, R&D Systems) and PE-conjugated anti-human IgG1 (Fc-specific, Southern Biotechnology, Birmingham, Alabama, USA) for 5 min at 37 °C. Next, cells were labelled with antibodies directed against CD45 (APC-Cy7,BD Bioscience), CD11b (FITC,BD Bioscience), F4/80 (efluor450, eBioscience), Gr-1 (AF700, eBioscience), and CXCR4 (APC, Biolegend). Binding of ICAM-1/Fc to neutrophils was measured by using a flow cytometer.

Activation of endothelial cells

To measure activation of cultured endothelial cells (bEnd.3, cultured in DMEM supplemented with 10% FBS), cells were seeded into 12-well plates and stimulated with either Alum crystals (10 µg, InvivoGen), TNF (100 ng, R&D Systems), or PBS as a negative control for 4 h at 37 °C. Cells were then immunostained using antibodies directed against ICAM-1/CD54 (AF488, Biolegend), VCAM-1/CD106 (Pacific blue, Biolegend), or E-selectin/CD62E (PE, BD Bioscience). After incubating the samples with lysing solution as described earlier and washing the samples twice in PBS, samples were resuspended in 200 µl PBS and analyzed *via* flow cytometry. The same protocol was used to assess the effects of DAMPs on endothelial cells.

Cell proliferation

Tumor cells were seeded on a 96-well plate and either treated with Alum crystals (10 µg) or MCC950 (10 µM). After 48 h, serum free media and the MTT reagent was added according to the manufacturers protocol (Abcam, Cambridge, United Kingdom) for 3 h at 37 °C. Next, MTT solvent was added and the plate was placed on an orbital shaker for 15 min. Finally, absorbance was measured at 590 nm in a microplate reader (Tecan, Männedorf,

Switzerland). Cell proliferation was determined as the percentage of change as compared to the negative control after background subtractions.

Furthermore, the effect of tumor-primed and control neutrophils on tumor cell or endothelial cell proliferation was investigated. Therefore, neutrophils from tumor bearing mice were isolated with the EasySep™ Mouse Neutrophil Enrichment Kit (STEMCELL Technologies, Vancouver, Canada), as described by the manufacturer. Isolated neutrophils from tumor-free (representing non-aged and physiologically aged neutrophils) or from tumor-bearing mice (exhibiting predominantly excessively aged neutrophils) were placed in the cell culture and incubated over night at 37 °C. On the following day, supernatants from the isolated neutrophils were placed onto tumor cells or endothelial cells. After incubation for 24 h at 37 °C, the MTT assay was performed.

Endothelial cell migration

Endothelial cells were seeded onto 6-well plates. After creating a “scratch” in the cell monolayer and washing the cells, supernatants from cultured tumor-primed and control neutrophils (obtained and isolated as described above) were added to the cultured endothelial cells. After 24 h, the number of cells migrated into the scratch region was assessed by light microscopy.

Mittmann LA, et al. J Immunother Cancer 2021; 9:e003495. doi: 10.1136/jitc-2021-003495

Supplementary Tables

mouse strain	tumor entity	model	day 0	day 14	day 14
**C3H**	**tumor free**		0.5 ± 0.1	-	-
	**SCC VII**	**orthotopic**	-	0.5 ± 0.1	0.7 ± 0.1
	**SCC VII**	**heterotopic**	-	0.5 ± 0.1	0.5 ± 0.1
**BALB/c**	**tumor free**		2.2 ± 0.4	-	-
	**4T1**	**orthotopic**	-	8.0 ± 2.4	26.0 ± 4.1
	**4T1**	**heterotopic**	-	3.9 ± 0.5	7.5 ± 2.6

Table S1. Systemic neutrophil counts in tumor-free and tumor-bearing mice. Numbers of neutrophils in the peripheral blood (in 10^3 μl^{-1}) of tumor-free or tumor-bearing (orthotopic or heterotopic) mice are given. Data are shown as mean±SEM.

see separate file

Table S2. Statistics. Statistical details including the number of experimental groups, the number of experiments per group, the statistical test employed and P values, as well as P values for normality and equal variance testing are shown for each dataset.

Supplementary Figures

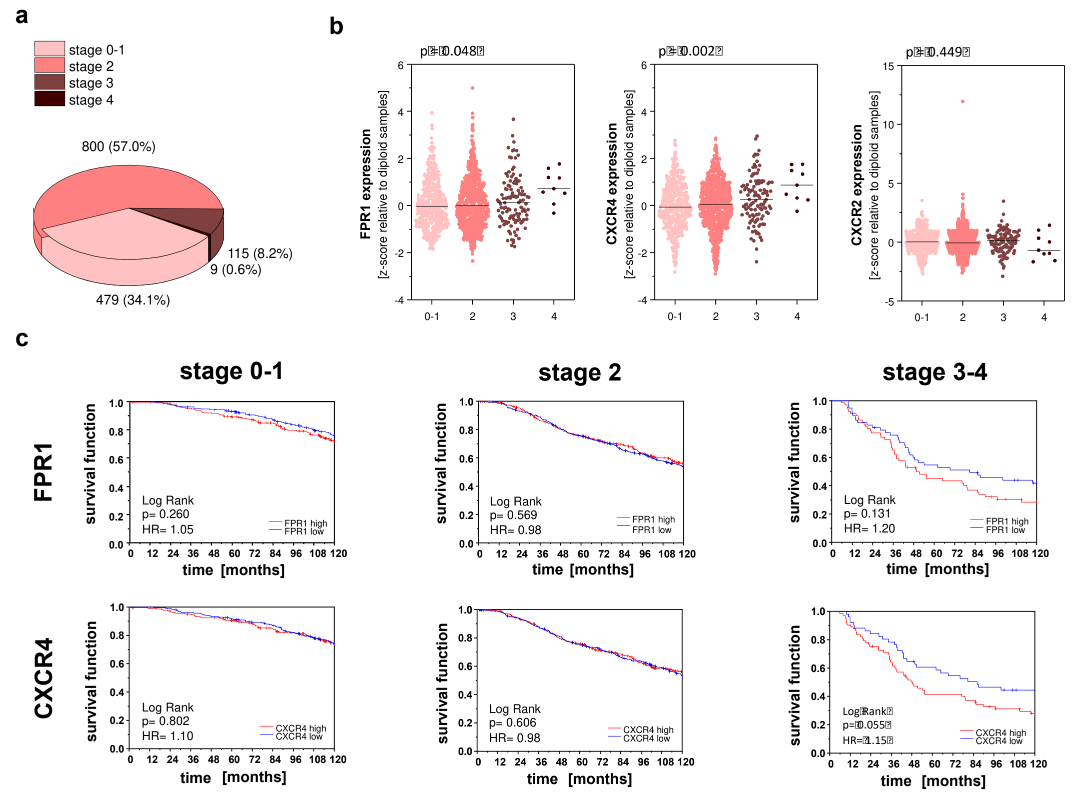

Figure S1. RNA expression of FPR1 and CXCR4 in human breast cancer. (**a**) Composition of analyzed patients of the METABRIC cohort with respect to the disease stage. (**b**) RNA expression of FPR1, CXCR2, or CXCR4 in different disease stages. (**c**) Overall survival of breast cancer patients with respect to FPR1 or CXCR4 RNA expression.

Mittmann LA, *et al. J Immunother Cancer* 2021; 9:e003495. doi: 10.1136/jitc-2021-003495

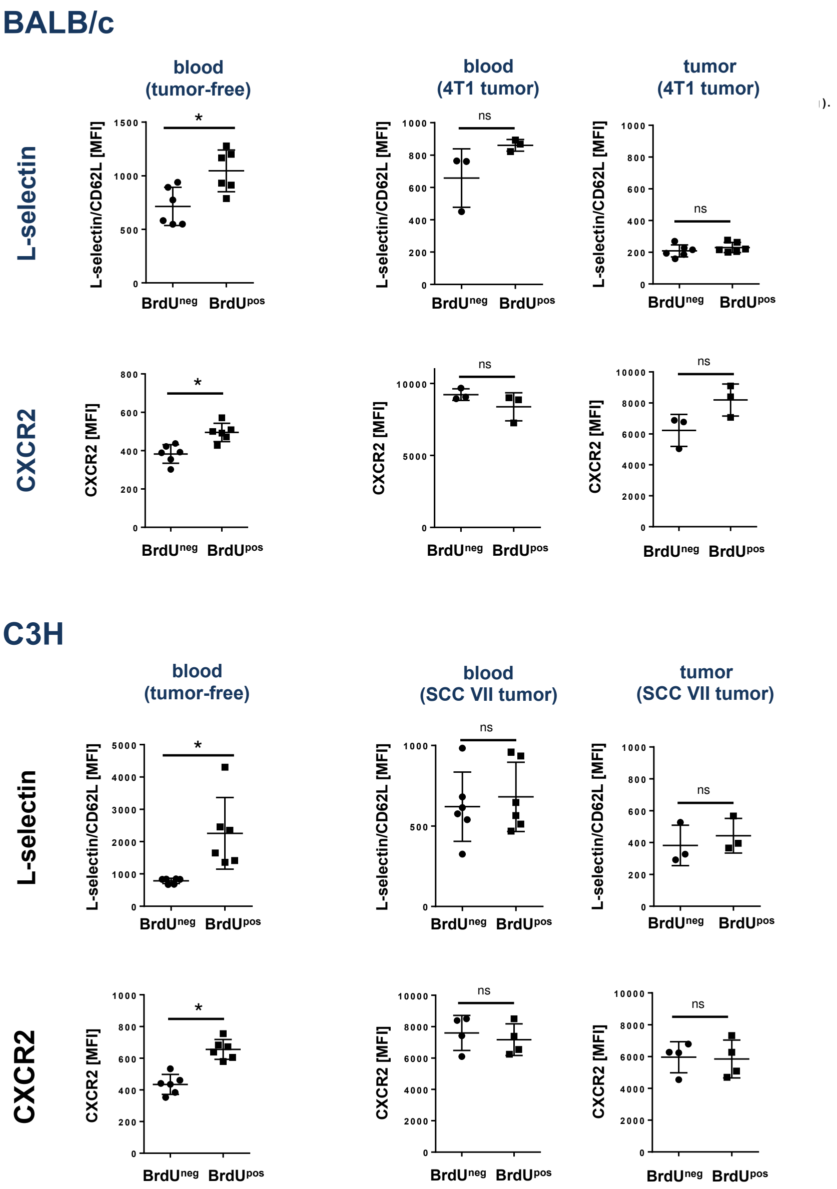

Figure S2. Surface expression of L-selectin and CXCR2 on aged and non-aged neutrophils. Surface expression of L-selectin/CD62L or the chemokine receptor CXCR2 on chronologically aged ($BrdU^{neg}$) and non-aged ($BrdU^{pos}$) neutrophils in the peripheral blood of tumor-free or of tumor-bearing C3H or BALB/c mice as well as of those recruited to tumors as assessed by multi-channel flow cytometry (n=3-6 mice per group). Data are shown as mean±SEM; *$p<0.05$ vs. $BrdU^{neg}$; ns=not significant.

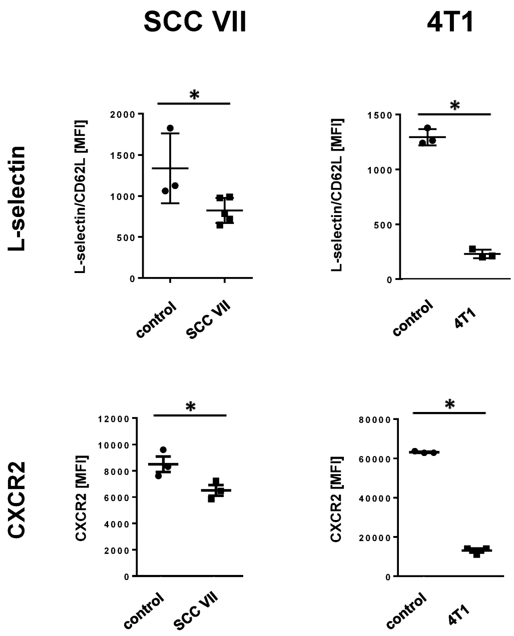

Figure S3. Average surface expression of L-selectin and CXCR2 on circulating neutrophils. The average surface expression of L-selectin/CD62L or the chemokine receptor CXCR2 on total circulating neutrophils in the peripheral blood of tumor-free or of tumor-bearing C3H (SCC VII tumors) or BALB/c (4T1 tumors) mice as assessed by multi-channel flow cytometry (n=3-5 mice per group). Data are shown as mean±SEM; *p<0.05 vs. control.

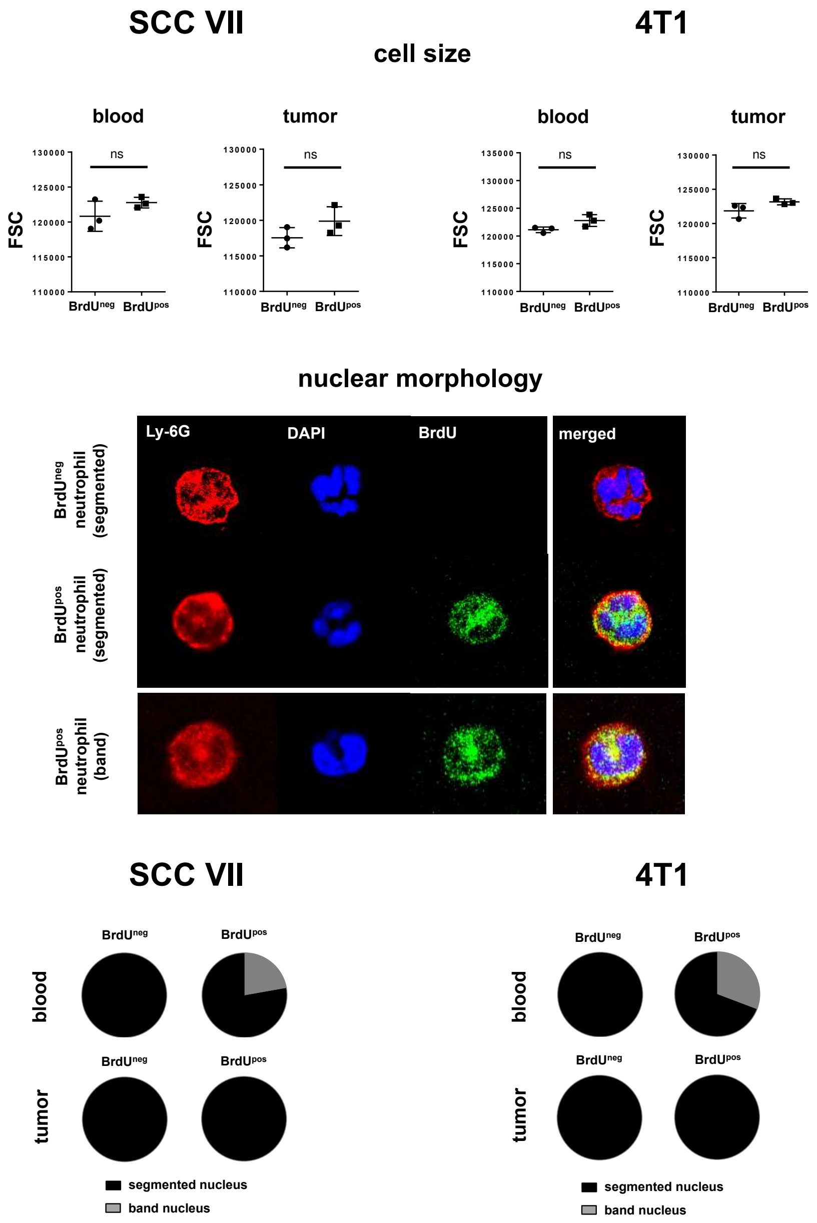

Figure S4. Size and nuclear morphology of neutrophils. Size (forward scatter; FSC) and nuclear morphology of aged ($BrdU^{neg}$) and non-aged ($BrdU^{pos}$) neutrophils as assessed in the peripheral blood of tumor-free and in peripheral blood or tumors of SCC VII or 4T1 tumor-bearing C3H or BALB/c mice by multi-channel flow cytometry or confocal microscopy (n=3 mice per group). Representative confocal microscopy images, quantitative data, or relative frequency (depicted in circles) are shown (mean±SEM; ns=not significant).

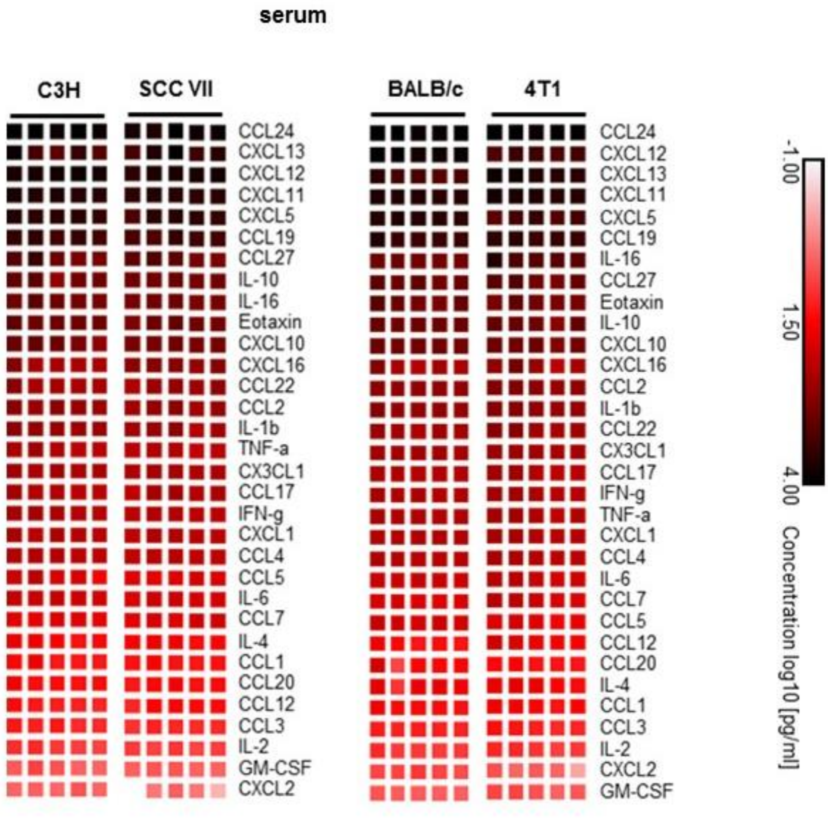

Figure S5. Serum cytokine levels in tumor-bearing mice. Expression patterns of cytokines in the serum of tumor-free and SCC VII or 4T1 tumor-bearing mice as assessed by multiplex ELISA analyses, data are shown as heatmaps (n=5 mice per group).

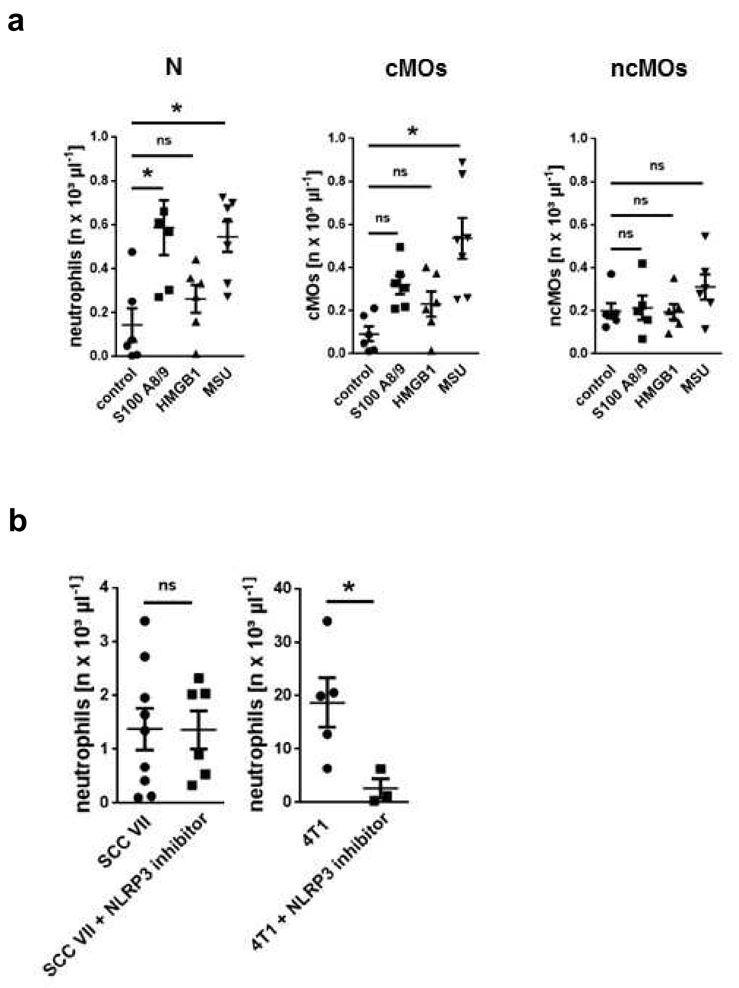

Figure S6. Effect of DAMPs on leukocyte trafficking. (**a**) Recruitment of neutrophils (N), classical monocytes (cMOs), and non-classical monocytes (ncMOs) into the peritoneal cavity 6 h after intraperitoneal injection of different DAMPs as assessed by multi-channel flow cytometry (n=6-7 mice per group). **(b)** Systemic neutrophil counts in tumor-bearing mice treated with a NLRP3 inflammasome inhibitor or vehicle as assessed by multi-channel flow cytometry (n=3-9 mice per group). Data are shown as mean±SEM; *p<0.05 vs. control/4T1; ns=not significant.

Mittmann LA, *et al. J Immunother Cancer* 2021; 9:e003495. doi: 10.1136/jitc-2021-003495

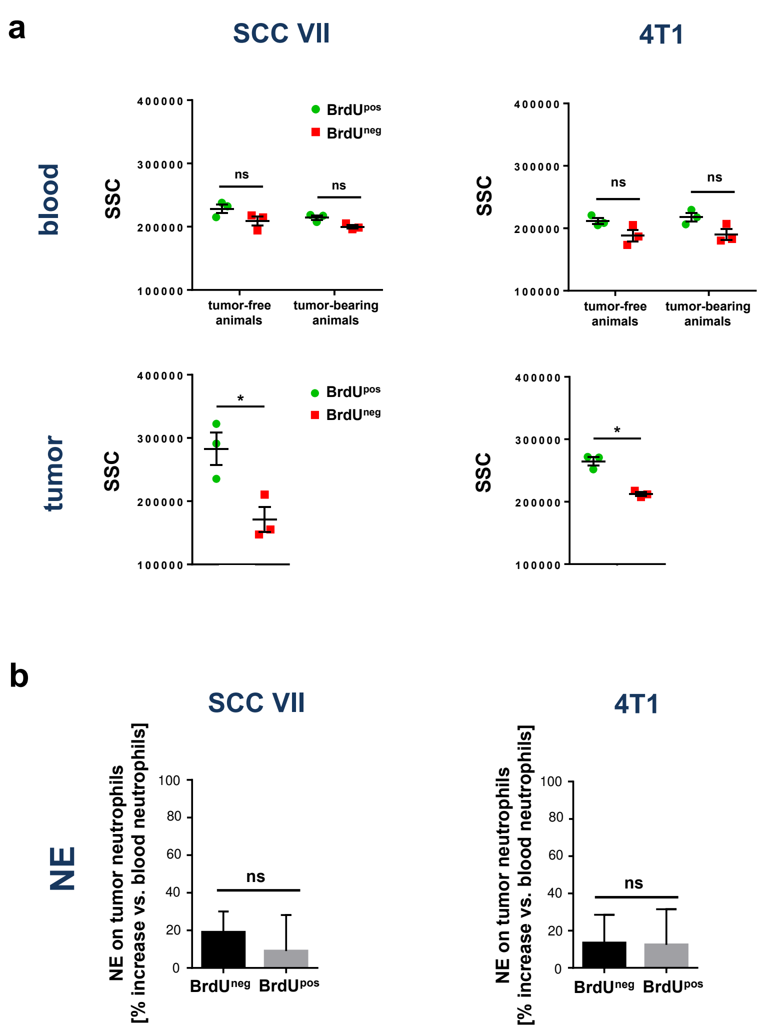

Figure S7. Degranulation of neutrophils during ageing in the circulation and in tumors. (**a**) Granularity (side scatter; SSC) of chronologically aged (BrdU^{neg}) and non-aged (BrdU^{pos}) neutrophils was assessed in the peripheral blood of tumor-free (control) as well as in the peripheral blood and in tumors of SCC VII or 4T1 tumor-bearing C3H or BALB/c mice by multi-channel flow cytometry (n=3 mice per group). (**b**) Relative change in surface expression of NE in BrdU^{neg} and BrdU^{pos} tumor neutrophils as compared to BrdU^{neg} and BrdU^{pos} blood neutrophils in tumor bearing mice. Data are shown as mean±SEM; *p<0.05 vs. BrdU^{pos}; ns=not significant.

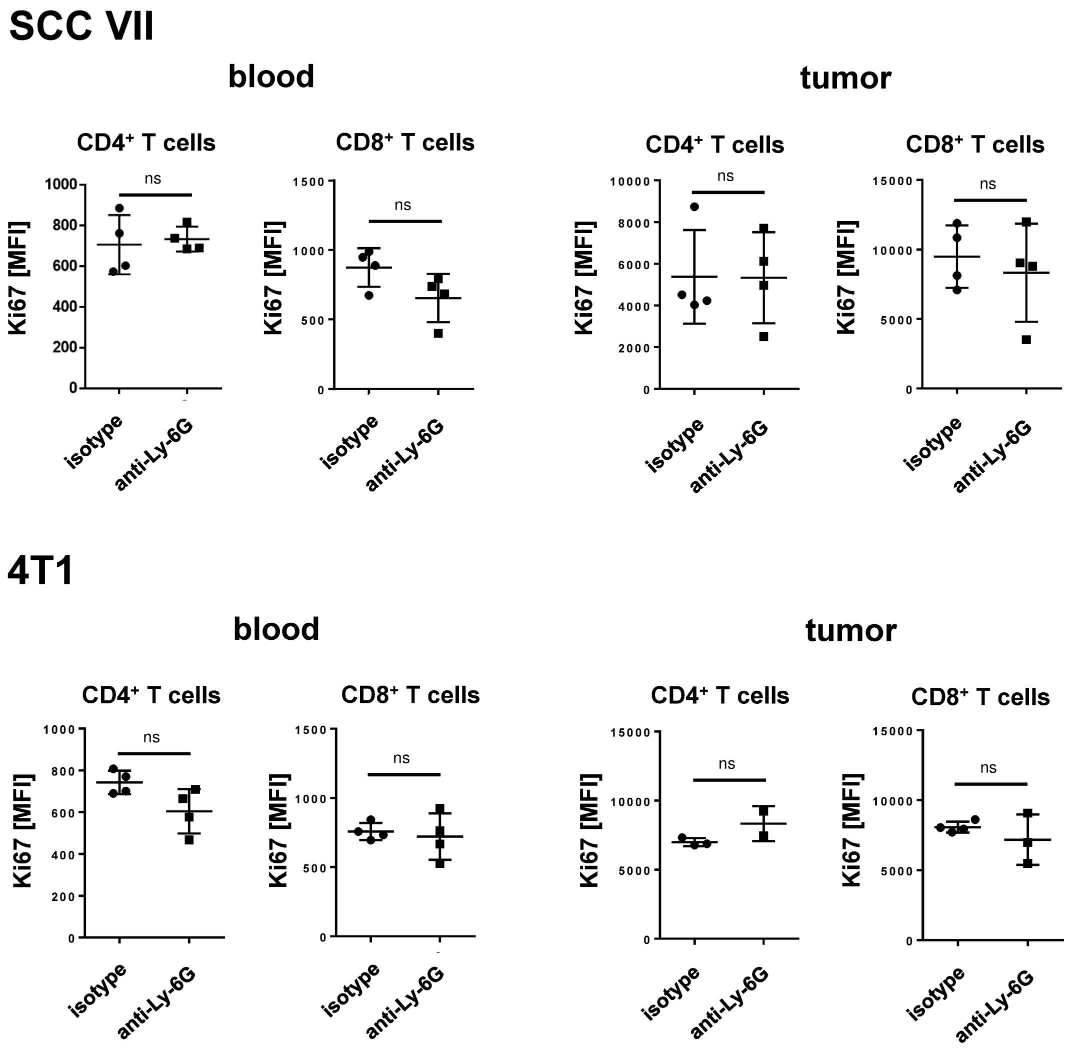

Figure S8. Effect of neutrophils on the proliferation of lymphocytes. As a measure of cell proliferation, expression of Ki-67 in CD4⁺ or CD8⁺ T cells in the peripheral blood or in tumors of mice with orthotopically grown SCC VII or 4T1 tumors receiving neutrophil-depleting anti-Ly-6G or isotype control antibodies was assessed by multi-channel flow cytometry (n=3-4 mice per group). Data are shown as mean±SEM; ns=not significant.

Supplementary References

1. Curtis C, Shah SP, Chin SF, et al. The genomic and transcriptomic architecture of 2,000 breast tumours reveals novel subgroups. *Nature* 2012;486(7403):346-52. doi: 10.1038/nature10983 [published Online First: 2012/04/24]

2. Gao J, Aksoy BA, Dogrusoz U, et al. Integrative analysis of complex cancer genomics and clinical profiles using the cBioPortal. *Science signaling* 2013;6(269):pl1. doi: 10.1126/scisignal.2004088

3. Casanova-Acebes M, Pitaval C, Weiss LA, et al. Rhythmic modulation of the hematopoietic niche through neutrophil clearance. *Cell* 2013;153(5):1025-35. doi: 10.1016/j.cell.2013.04.040

4. Baez S. An open cremaster muscle preparation for the study of blood vessels by in vivo microscopy. *Microvascular Research* 1973;5(3):384-94. doi: https://doi.org/10.1016/0026-2862(73)90054-X

5. Schindelin J, Arganda-Carreras I, Frise E, et al. Fiji: an open-source platform for biological-image analysis. *Nature Methods* 2012;9:676. doi: 10.1038/nmeth.2019

https://www.nature.com/articles/nmeth.2019#supplementary-information

6. Uhl B, Vadlau Y, Zuchtriegel G, et al. Aged neutrophils contribute to the first line of defense in the acute inflammatory response. *Blood* 2016;128(19):2327-37. doi: 10.1182/blood-2016-05-718999 [published Online First: 2016/09/10]

7. Vogl T, Eisenblätter M, Völler T, et al. Alarmin S100A8/S100A9 as a biomarker for molecular imaging of local inflammatory activity. *Nature communications* 2014;5:4593-93. doi: 10.1038/ncomms5593
